# Magnetoelectric BAW and SAW Devices: A Review

**DOI:** 10.3390/mi15121471

**Published:** 2024-12-03

**Authors:** Bin Luo, Prasanth Velvaluri, Yisi Liu, Nian-Xiang Sun

**Affiliations:** Electrical and Computer Engineering Department, Northeastern University, Boston, MA 02115, USA; luo.bin1@northeastern.edu (B.L.); p.velvaluri@northeastern.edu (P.V.); liu.yisi@northeastern.edu (Y.L.)

**Keywords:** magnetoelectric (ME) devices, bulk acoustic wave (BAW), surface acoustic wave (SAW), magnetic field sensors, antennas, isolators, bio-sensing, wireless communication, quantum technology

## Abstract

Magnetoelectric (ME) devices combining piezoelectric and magnetostrictive materials have emerged as powerful tools to miniaturize and enhance sensing and communication technologies. This paper examines recent developments in bulk acoustic wave (BAW) and surface acoustic wave (SAW) ME devices, which demonstrate unique capabilities in ultra-sensitive magnetic sensing, compact antennas, and quantum applications. Leveraging the mechanical resonance of BAW and SAW modes, ME sensors achieve the femto- to pico-Tesla sensitivity ideal for biomedical applications, while ME antennas, operating at acoustic resonance, allow significant size reduction, with high radiation gain and efficiency, which is suited for bandwidth-restricted applications. In addition, ME non-reciprocal magnetoacoustic devices using hybrid magnetoacoustic waves present novel solutions for RF isolation, which have also shown potential for the efficient control of quantum defects, such as negatively charged nitrogen-vacancy (NV^−^) centers. Continued advancements in materials and device structures are expected to further enhance ME device performance, positioning them as key components in future bio-sensing, wireless communication, and quantum information technologies.

## 1. Introduction

Over the past few decades, magnetoelectric (ME) devices, which are based on a combination of both piezoelectric and magnetostrictive materials, have demonstrated their potential to miniaturize devices while enhancing functionality. In 2001, ME composites made of bi-layer laminates were demonstrated, marking a significant development in the field [1,2]. Since then, numerous ME composite materials and devices have been reported for various applications [3,4].

The efficiency of ME materials primarily depends on the magnetoelectric coefficient (α); a higher α indicates a greater response to stimuli [5]. This coefficient is constrained by the quality of the individual materials and the degree of connectedness between them. For a given composite material, α can be enhanced when measured at the mechanical resonance of the device, where it is amplified by the quality factor. Mechanical resonance is typically achieved by exciting the piezoelectric material, thereby generating a bulk acoustic wave (BAW) within the device. Numerous BAW-based ME devices, such as sensors and antennas, have been extensively investigated.

ME magnetic sensors capable of detecting bio-magnetic fields in the pico-Tesla to femto-Tesla range at room temperature have been demonstrated [6], offering an attractive alternative to other magnetic sensor techniques, such as magnetoencephalography (MEG), magnetocardiography (MCG), magnetomyography (MMG), and magnetoneurography (MNG) [7,8,9]. Their ultra-compact design makes them ideal for having higher spatial resolution. Cantilever-based magnetic field sensors towards biosensing using an inverse ME magnetoelectric effect are well investigated [10,11,12], with recent developments including an exchange bias-based magnetic multi-layer structure demonstrating a limit of detection (LOD) of 18 pT/Hz^1/2^ at 10 Hz [13]. More compact designs using nano-plate resonators capable of simultaneous magnetic field sensing and energy harvesting have also been demonstrated [14].

ME antennas based on BAW resonators, operating at their mechanical resonance, present a promising alternative to conventional electromagnetic antennas, which operate at electromagnetic resonance [15,16,17]. Operating at acoustic resonance and utilizing magnetic radiation allow these antennas to be significantly miniaturized without ground plane effects [18], making them highly useful for applications with strict size constraints, such as microwave devices and medical implants. Various ME antenna designs are discussed in the literature, each with its own advantages and limitations. Recent advancements include antenna arrays for increased gain [19] and solidly mounted resonators for improved power-handling capacity and stability [20].

Similarly, surface acoustic waves (SAW) can also be excited in piezoelectric substrates or layers, enabling a range of applications. SAW-based sensors employ various working principles, such as mass loading for bio-detection or the delta-E effect [21,22,23,24] for magnetic field detection. In these cases, a SAW generated at one end travels to the other, with an active layer between the delay line on the sensor substrate. The active layer changes the velocity of the traveling wave as a function of a particular stimulus (e.g., magnetic field), and this causes a phase shift in the traveling wave. Given the range of SAW devices, this review focuses on those using ME materials, including applications in magnetic field sensors, antennas, and isolators. SAW-based magnetic field sensors are well-studied in the literature, with an emphasis on detecting pico-Tesla-level magnetic fields [25]. Their high bandwidth makes them ideal candidates for bio-magnetic sensing [22]. An exchange-biased SAW magnetic field sensor with reduced magnetic noise achieves an improved LOD down to 28 pT/Hz^1/2^ at 10 Hz and 10 pT/Hz^1/2^ at 100 Hz [25], and novel SAW-based ME antennas have also been reported.

**Figure 1:**
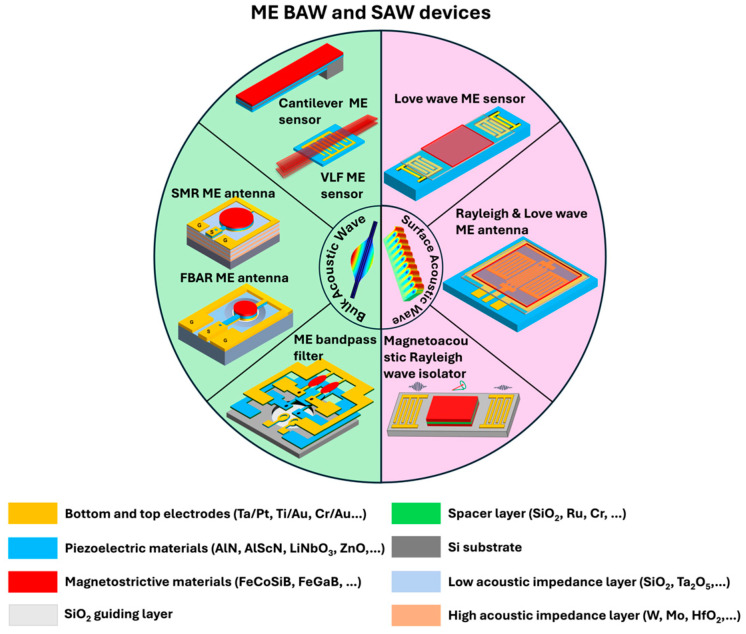
Magnetoelectric (ME) device possibilities using BAW and SAW concepts. The ME devices include magnetic sensors, antennas, isolators, and filters.

In this work, we start with a foundational overview of BAW and SAW concepts and a brief overview of the typical ME materials and their critical properties. After this, we present recent advancements in BAW and SAW technology in ME applications, including magnetic field sensors, ultra-high and low-frequency antennas, and isolators (Figure 1).

## 2. Magnetoelectric Materials for BAW and SAW Devices

### 2.1. BAW and SAW

Bulk acoustic wave (BAW) and surface acoustic wave (SAW) devices have been widely used in sensors, actuators, filters, and oscillators, among others [26,27]. For instance, SAW actuators can repair or quarantine damage in mechanical systems for structural health monitoring [28] and transport material on microscales, and even nanoscales [29]. On the other hand, SAW sensors play a significant role in the chemical detection of explosives and vapors [30,31,32] and the biosensing of cancers, DNA, antigens–antibodies, biotoxins, etc. [33,34,35]. The performance of magnetoelectric devices highly depends on the mechanical quality factor of the acoustic resonators (the ratio of stored energy to energy loss in one resonant period), mechanical loss, and dielectric loss of wave modes. The parameters of the BAW and SAW modes used in ME devices are summarized in Table 1.

BAW is generated by applying an alternating AC voltage on the top and bottom of a piezoelectric layer. Due to the piezoelectric effect, a standing BAW can be built up in the thickness direction. Depending on the polarization of displacement, the BAW can be categorized into thickness-extensional mode with out-of-plane polarization and thickness-shear mode with in-plane polarization [36]. The resonance frequency is inversely proportional to the resonator thickness, where the wavelength equals twice the thickness. A thinner piezoelectric layer leads to a higher operation frequency. Typical BAW resonators include thin-film bulk acoustic resonators (FBAR) and solidly mounted resonators (SMR). FBAR is suspended in the air to reduce acoustic loss and enhance the mechanical quality factor. The suspension of the resonator can be realized by etching the backside of the Silicon on Insulator (SOI) wafer or etching the Si substrate with XeF_2_ vapor. Even though FBAR shows an extremely high mechanical quality factor and can be realized by releasing the resonator from a substrate [37], it is fragile and suffers from a low power-handling capability [20]. Compared to FBAR, SMR utilizes an acoustic Bragg reflector composed of several periods of low impedance (e.g., SiO_2_, Ta_2_O_5_) and high impedance layers (W, Mo, etc.) to confine the acoustic energy in the acoustic resonator [38]. SMR shows an advantage of strong power-handling capability [20] but typically has a degraded mechanical quality factor owing to additional acoustic energy leakage and loss brought about by the reflector [39]. In addition, the fabrication of such a resonator is complex, and the control of the surface roughness is crucial to reducing acoustic loss and maintaining the magnetic properties of the magnetostrictive film on top [40].

SAW devices are composed of two pairs of identical receiving and transmitting interdigital transducers (IDTs) on a piezoelectric film (e.g., AlN film on Si) or substrate (e.g., bulk single-crystal 128 Y-X cut LiNbO_3_). By applying a RF voltage on the transmission (Tx) IDTs, a surface acoustic wave can be generated to propagate towards the receiving (Rx) IDTs. The displacement magnitude decreases exponentially inside the piezoelectric materials, and most of the acoustic energy concentrates on the surface. Based on whether acoustic Bragg reflectors are used, SAW devices can be categorized into SAW delay lines and SAW resonators. Reflectors can be made of metal or dielectric stripes, grooves, and ion-implanted stripes [3]. SAW delay lines have a transmission behavior like a sinc function $\;\frac{\sin\left(N\pi \left(f-f_{0}\right)T\right)}{N\pi \left(f-f_{0}\right)T}$, where *N*, *f*_0_, and *T* refer to the number of finger pairs, center frequency, and acoustic travel time between the two IDTs, respectively. SAW delay lines have a relatively wide bandwidth but exhibit a high insertion loss due to acoustic energy leakage on the two sides of IDTs [41]. The bandwidth of the SAW delay lines is inversely proportional to the IDT finger numbers. With the depositions of additional Bragg reflectors, a standing wave can be formed inside the cavity of the SAW resonator, leading to a high mechanical quality factor with one sharp transmission peak. Typically, the pitch of Bragg reflectors is designed to be identical to that of the receiving and transmission IDTs [41]. Compared to BAW, the resonance frequency of the SAW devices was determined by the IDT design. The design formula of the resonance frequency, acoustic wavelength, and IDT pitch size (distance between the center of the two fingers) can be found in Table 1. One big challenge of SAW devices above 3 GHz is the increase in acoustic and electrical loses. In addition, the high frequency needs narrow IDTs that cannot be fabricated by industrial photolithography and require the use of laser or e-beam photolithography. The weak power handling of the narrow IDTs make their use challenging for practical applications.

**Table 1:** BAW and SAW modes in ME devices.

SAW/SAW Mode	BAW Thickness Extensional Mode	SAW Rayleigh Wave	SAW Love Wave
Resonator structure and acoustic wave profile	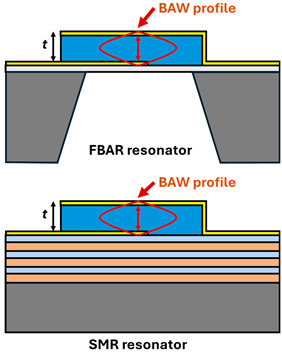	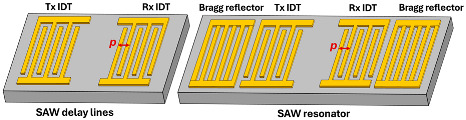	
		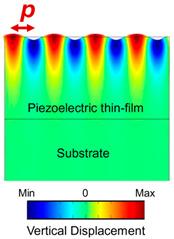	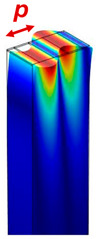
Wavelength	λ=2t	λ=2p $w_{IDT,\;single}=\frac{\lambda }{4}$ $w_{IDT,\;double}=\frac{\lambda }{8}$	λ=2p $w_{IDT,\;single}=\frac{\lambda }{4}$ $w_{IDT,\;double}=\frac{\lambda }{8}$
Resonance frequency	$f_{r}=\frac{v_{t}}{2t}=\frac{1}{2t}\sqrt{\frac{E_{eq}}{\rho_{eq}}}$	$f_{r}=\frac{c_{R}}{\lambda }$	$f_{r}=\frac{c_{L}}{\lambda }$

where *p*: pitch size; *λ*: wavelength; *t*: thickness of the BAW resonator, *c_R_*: Rayleigh wave velocity; *c_L_*: Love wave velocity; *E_eq_*: equivalent Youngs modulus; *ρ_eq_*: equivalent mass density. Here we assume the metallic ratio equals 0.5, which is used most commonly. The SAW Rayleigh wave profile is produced with permission from Ref. [3]. 2022 AIP Publishing. The SAW Love wave profile is produced with permission from [42].

### 2.2. Magnetoelectric Composites and Materials

#### 2.2.1. ME Composites

Magnetoelectric (ME) materials are a special class of materials that can possess more than one ferroic property. Such materials can simultaneously exhibit ferroelectric and ferromagnetic properties. The direct ME effect corresponds to a change in electrical polarization in the material when it is subjected to a change in magnetic field, while the converse effects correspond to a change in magnetization upon changing the electrical field. The ferroelectric property of the materials is due to the relative shifts between the negative and positive charges in the materials, which usually require empty d orbitals. On the other hand, the ferromagnetic effect originates from the spins of the partially occupied d orbitals in the material. Due to these contradicting requirements, there are very limited naturally occurring single-phase magnetoelectric materials. Though such materials were postulated in 1894 [43], the first experimentally reported single-phase ME material was Cr_2_O_3_ in 1960 [44]. Other single-phase bulk ME materials include BiFeO_3_ [45,46,47], Aurivillius phases [48,49,50], Hexaferrite (Sr_3_Co_2_Fe_24_O_41_) [51], and layered perovskites (YBa_1-x_Sr_x_CuFeO_5_) [52]. Though few of these materials demonstrate the room temperature bulk ME effect, only BiFeO_3_ is considered for actual applications [45,46,47].

Alternatively, multiferroic ME materials have a combination of inorganic ferroelectric (piezoelectric) and ferromagnetic (magnetostrictive) materials. This approach has an added advantage in that one could optimize/tailor the individual layer for the applications. Thus, it gives a higher degree of freedom for material selection. Direct piezoelectric materials produce an electrical charge when subjected to mechanical stress. The magnetostrictive materials produce a strain in the material when subjected to a magnetic field. When both these materials are coupled, then such a composite could exhibit a composite ME effect. In such materials, the ME coefficient depends on the following parameters.

$$\alpha =d\cdot d^{m}\cdot k_{c}=\frac{\partial P}{\partial H}$$

In the above equation, α is the ME coefficient, which gives the change in polarization due to the change in the magnetic field; $d=\frac{\partial P}{\partial S}$ is the piezoelectric coefficient, which maps the change in polarization (P) due to the strain (S) in the material. $d^{m}=\frac{\partial S}{\partial H}$ is the piezomagnetic coefficient, and it gives the change in strain (S) in the materials for a change in the magnetic field (H). $k_{c}$ is the coupling coefficient, which varies from zero to one and depends on the elastic connectivity between both materials.

In the case of bulk materials, the race to get composites with a high α was initiated by combining particulate mixtures of piezoelectric materials (PbZrTiO_3_, BaTiO_3_, and PbTiO_3_, among others) and combining with ferrites (NiFe_2_O_4_ and CoFe_2_O_4_, among others). Such composites exhibit α in the range of 1–500 mV/cm Oe [53,54,55,56,57]. The effect can be enhanced when it is measured at the mechanical resonance. This showed an increase in α to 6–7 V/cm Oe [58,59,60,61]. Another well-explored bulk composites configuration is the 2-2 laminates, where laminates comprising ferromagnetic materials with giant magnetostriction, such as Terfenol-D [62,63] or amorphous Metglas [64,65], were used in combination with PZT. In such composites, a high α~5–6 V/cm Oe is demonstrated without exciting the composites at their resonance.

With the onset of advances in microsystem technology in the early 2000s, the field of ME composites not only invented novel composites with a high α but also numerous real-world devices, such as magnetic field sensors, antennas [15], energy harvesters, isolators, and so on. The most well-researched connectivity explored is the 1-3 nanocomposites, where the 1D nanomagnetic pillars are embedded in a piezoelectric matrix, and 2-2 bilayer composites, where both are 2D films on a substrate. The former 1-3 connectivity is supposed to have a higher α due to its increased surface area between the ferroelectric and magnetic phases [66,67]. However, leakage currents in the magnetic pillars limit their α to between 20 and 80 mV/cm Oe [68,69,70]. On the other hand, the 2-2 laminar composites, initially thought to suffer from substrate clamping, have shown promising results, e.g., AlN/FeCoBSi [71] and BaTiO_3_/CoFe/BaTiO_3_ [72] have shown α = 3–5 V/cm Oe due to perfect coupling. A further enhancement of α in AlN/FeCoBSi composites to 9.7 kV/cm Oe at resonance in air and 19 kV/cm Oe at resonance under vacuum was demonstrated [73]. The 2-2 laminar configuration is most used in the BAW resonators.

#### 2.2.2. Piezoelectric Materials

The piezoelectric materials were first discovered by Pierre and Jacques Curie in 1880 when they saw that certain crystals created an electrical charge when they were subjected to mechanical compression. Only a year later, in 1881, Gabriel Lippmann predicted the inverse piezoelectric effect based on the fundamental thermodynamic principle, which was experimentally demonstrated later by the Curie brothers. The piezo effect occurs in materials that do not have a center of symmetry. In such materials, without any external stress, the pseudo-positive and the pseudo-negative centers inside the materials coincide. However, upon deforming the material, the pseudo-positive and -negative centers are separated from each other, thus, resulting in creating a dipole movement in the crystal due to the application of mechanical stress. The fundamental equations that govern the direct and indirect piezo effect are as follows.

Direct piezo effect:D=ε·E+d·σ

$$For\;indirect\;piezo\;effect:S=s^{E}\cdot \sigma +d\cdot E$$

where E is the electric field, D is the dielectric displacement, σ is the stress, and S is the strain field tensors. The ε is the dielectric permittivity, which is a (3 × 3) matrix, while the $s^{E}$ is the compliance matrix (6 × 6) (which is the inverse of the stiffness matrix). d is the piezoelectric coefficient, and it is a (3 × 6) matrix. If d=0, the equations for the direct effect transform into an equation describing electrical materials, while the indirect piezo effect transforms into Hooke’s law, which relates mechanical stress with strain. The piezoelectric matrix is further expanded as:

$$d=\left(\begin{array}{ccc} d_{11} & \cdots & d_{16}\\ \vdots & \ddots & \vdots \\ d_{31} & \cdots & d_{36} \end{array}\right)$$

The most important parameters in the matrix are the $d_{31},\;d_{32}$ and $d_{33}$ elements. The $d_{33}$ is the parameter of interest when the piezoelectric material is operated longitudinally while the $d_{31},\;d_{32}$ are important during transverse operations.

With respect to the BAW and SAW devices, piezoelectric materials, whether single crystals or polycrystals, play a crucial role in acoustic wave excitation. In such devices, the piezoelectric materials are supplied with a RF voltage, which can excite an acoustic wave in the material. Shown in Table 2 is the list of commonly used piezoelectric materials for BAW and SAW devices. Though materials like PZT, PMN-PT, and PZN-PT possess high $d_{33},$ they have high losses and are not CMOS compatible. Alternatively, materials like quartz, LiNbO_3_, and LiTaO_3_ are well-used for realizing SAW/BAW devices, owing to their low losses and CMOS compatibility.

#### 2.2.3. Magnetostrictive Materials

The discovery of magnetostriction predates the piezoelectric effect. It was discovered by James Prescott Joule in 1842 [126], when he observed a change in the dimension of iron when he altering its magnetization. The opposite effect, the Villari effect, was discovered in 1864 and showed a change in magnetization when there is stress applied to the material [127]. The magnitude of the dimensional change is represented by lambda (λ), which is the ratio of change in length to the original length. λ  is a dimensionless number and is typically reported in ppm. The origin of the effect is in the interaction of atomic magnetic moments with the elastic bond lengths of the crystal.

Above the Curie temperature, all the magnetic moments in a ferromagnetic material do not have any magnetic order, and the net magnetization is equal to zero. Upon cooling, spontaneous magnetization occurs in the material, and all of the magnetic moments align along the easy axis of the material (due to anisotropy). The sample either shrinks or expands depending on the sign of the magnetostriction constant, as the magnetic moments rotate towards or away from the external magnetic field direction. Now, if an external magnetic field is applied, such that the magnetic domains rotate 90 degrees, then one can get maximum magnetostriction. However, if the magnetic field is applied, such that the magnetic moments rotate by 180 degrees, then there is no effective change in the shape of the sample [128]. The change of magnetostriction as a function of the magnetic field, $d^{m}=\frac{\partial \lambda }{\partial H}$, is of interest and is termed the piezomagnetic coefficient. λ increases/decreases as a function of the external field and saturates when all of the magnetic moments point in the direction of the external magnetic field. This is termed saturation magnetostriction, $\lambda_{s}$. The equations that govern the magnetostriction are given by:

$$B=\mu^{\sigma }\cdot H+d^{m}\cdot \sigma$$

$$S=s^{H}\cdot \sigma +d^{m}\cdot H$$

where B is the magnetic induction, H is the magnetic field, and σ is the stress field tensors. $\mu^{\sigma }$ is the permeability at constant stress. $s^{H}$ is the compliance, and $d^{m}=\frac{\partial \lambda }{\partial H}$ is the piezomagnetic coefficient of the material. In the above equations, if there is no magnetostriction ($d^{m}=0$), the equations transform to describe classic magnetic materials and stress with strain (Hooke’s law) relation.

Rare earth-based bulk materials exhibit the highest magnetostriction. This is mainly due to the partially filled 4f orbitals, which have strong anisotropy [129]. Giant magnetostrictions of around 6000 ppm in Dysprosium [130] and 3000 ppm in Terbium [131] were reported in the 1960s, but the Curie temperature of these materials is well below room temperature (RT).

The highest RT magnetostriction achieved by far in either bulk or thin films is Terfenol-D (Tb_0.27_Dy_0.73_Fe_2_). It can demonstrate a $\lambda_{s}$ of around 1840 ppm. It is obtained by combining two different Laves phase materials, TbFe_2_ and DyFe_2_, with opposite anisotropies, which have near-zero magnetic anisotropy [132,133]. Other materials that exhibit high magnetostriction are Galfenol (Fe_81_Ga_19_) [134] and FeSiBMn (Metglas) [135].

In thin films, giant magnetostriction was first reported by Quandt et al. in TbFe/FeCo multilayers with a saturation field of 20 mT [136,137]. Materials with such low saturation fields have high industrial relevance. In recent years, increasing studies has focused on actual devices, such as magnetic sensors, RF filters, and antennas, where materials with high piezomagnetic coefficients $\left(\frac{\delta \lambda }{\delta H}\right)$ are particularly important. Thin film magnetostrictive materials have a high piezomagnetic coefficient, mainly originating from the absence of magnetocrystalline anisotropy. A few examples are FeGaB, FeCoBSi, CoFeC, and FeGaC. Table 3 below summarizes the bulk and thin film magnetostrictive materials and their parameters.

## 3. Magnetoelectric Sensors

Magnetic sensors are critical components in a wide range of technological applications due to their ability to detect and measure weak magnetic field signals with high sensitivity for high-precision measurements. They are crucial in industrial automation, navigation systems, medical diagnostics, and scientific research. For instance, in biomedical applications, magnetic sensors enable non-invasive, contactless, flexible, and fast detection of weak bio-magnetic signals from human organs and tissues, such as nerves, muscles, brains, and hearts, making them vital in health monitoring and medical diagnosis. Existing bio-magnetic sensing and diagnosis techniques, including magnetic resonance imaging (MRI) [146], magnetoencephalography (MEG), magnetocardiography (MCG) [7,147], magnetomyography (MMG), and magnetoneurography (MNG) [8,9], have provided real-time insight into physiological functions like neural and cardiac activities for invaluable data in both research and clinical environments. In industrial applications, magnetic sensors are employed for current sensing, position tracking, and detecting flaws in materials. They are essential for precision control and feedback systems in robotics and automated machinery, offering robustness in harsh environments like high temperatures and humidity [148]. Magnetic sensors are used for contactless current, angular position, and switch sensing in green energy power plants such as wind turbines and solar panel farms for optimal wind power generation. Their applications continue to expand as they become more integrated with emerging technologies like IoT and Industry 4.0 [149,150], further underscoring their importance.

Over the decades, various magnetic sensors have been intensively investigated and developed, aiming to improve sensitivity and reduce size, especially for bio-magnetic applications requiring stable fT-level magnetic sensing over a broad frequency range from 0.01 Hz to 1000 Hz [151,152,153,154]. Superconducting quantum interference devices (SQUID) are state-of-the-art magnetometers with a fT range magnetic signal sensing capability [155], which makes them capable of monitoring brain, nerve, skeletal muscle, and heart activities. However, the requirement for a cryogenic environment provided by liquid helium makes the operation of SQUID expensive, bulky, and energy intensive. Alternative systems, such as optically pumped magnetometers, also have similar issues of high complexity and cost owing to the need for a laser and a gas chamber, despite a superior equivalent magnetic noise level of below 100 fT/√Hz [156,157,158,159,160,161,162]. The miniaturization of magnetic sensors has been realized by advanced micro–nano fabrication techniques, leading to the demonstration of giant magnetoimpedance (GMI) sensors [163,164,165,166,167,168], giant magnetoresistance (GMR) [169,170,171] and tunnel magnetoresistance (TMR) sensors [172,173], etc. However, the notable thermal, flicker-, and spin–torque noise originating from high driving current limit the sensing performance of these compact magnetic sensors to the pT/Hz^1/2^ level [174,175,176,177]. The need for miniaturization, low noise, low power, and extremely high sensitivity has driven the development of the bulk acoustic wave (BAW) magnetoelectric (ME) sensors [11,147,178,179,180,181,182,183,184,185,186,187,188], which utilize high quality factor (Q) magnetoelectric coupling in a ME composite and realize strain-mediated power-efficient conversion from weak magnetic signals to voltage outputs. On the other hand, the surface acoustic wave (SAW) sensor can detect ultra-sensitive magnetic signals owing to the significant transmission change between SAW transducers in response to the modulus change of magnetostrictive film [22,23,24,25]. The state-of-the-art magnetoelectric magnetometers can enable the detection of single-digit fT magnetic fields at kHz at room temperature, greatly lowering the cost, power consumption, and form factor for biomedical magnetometry systems [4,189]. The following subsections will review the recent progress of BAW and SAW ME sensor fabrication, material synthesis, modulation scheme, and detection and noise performance improvement.

### 3.1. BAW ME Sensor

#### 3.1.1. Bulk ME Sensors

The BAW ME sensors utilize the direct ME effect to detect the voltage output in the piezoelectric layer induced by the strain of the magnetostrictive layer under an external magnetic field signal [4,190,191]. Depending on the sizes of the piezoelectric phase and the magnetostrictive phase, they can be categorized into bulk and thin-film BAW ME sensors. The bulk ME sensors are typically composed of ME laminates with one single-crystal piezoelectric substrate, such as PZT and PMN-PT, sandwiched by an epoxy-glued magnetic foil like Metglas [192]. Depending on the orientation of magnetization in the magnetostrictive layer and polarization in the piezoelectric layer, the bulk ME sensors can operate on different combined modes, including L-L, L-T, T-T, and T-L, where T refers to transverse and L stands for longitudinal. Since the thickness of the magnetostrictive is typically much smaller than the lateral dimension, the strong demagnetizing field forces the magnetostrictive to have in-plane magnetization. Therefore, most bulk ME sensors work on L-L or L-T modes so that they can exhibit a high ME coupling coefficient α_ME_ for high magnetic field sensitivity. Since α_ME_ can realize a 100 times improvement at electromechanical resonance (EMR) [193], the direct detection scheme of the ME voltage at EMR has been developed, with intensive efforts in boosting α_ME_ via geometry optimization [194,195,196,197,198], novel material development [199,200], and sensor fabrication improvement [201,202,203]. To reduce the hysteresis loss and enhance the mechanical quality factor of the ME resonator, Chu et al. utilized laser treatment on Metglas layers and epoxy-glued them to PMN-PZT fiber to form a ME sensor. The fiber can concentrate magnetic flux in the magnetic layer, leading to a high α_ME_ of 7000 V/cm Oe. By doping Mn into PMN-PZT, α_ME_ can be further boosted to 12,500 V/cm Oe owing to a more stabilized ferroelectric domain and pinned domain wall motion. However, the highest ME coefficient of bulk ME sensors is typically realized at tens of kHz or even higher. This frequency range is beyond the typical magnetic signal frequency from 0.01 Hz to 1 kHz.

**Figure 2:**
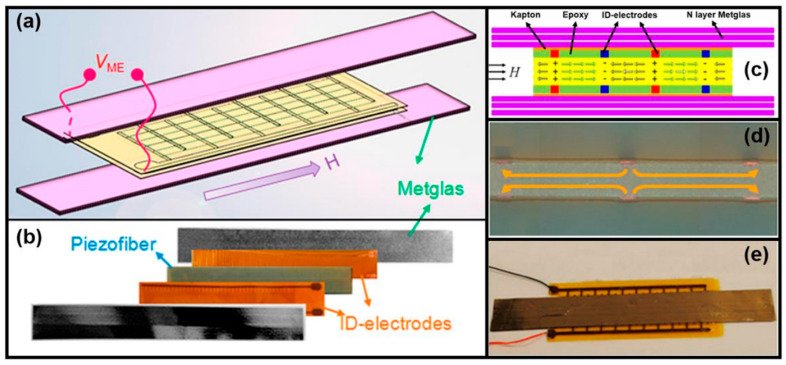
(**a**) Schematic diagram of the Metglas/piezo fiber ME sensor configuration, featuring ID electrodes on the PMN-PT fiber core composite and symmetric three-layer Metglas actuators. (**b**) Exploded view of individual components. (**c**) Illustration of multiple alternating push–pull units for enhanced ME coupling. (**d**) Optical microscopy image of a longitudinally poled push–pull element within the core composite. (**e**) Photographs of the fully assembled Metglas/piezo fiber ME sensor. Reproduced with permission from Refs. [17,64]. Copyright 2021 IEEE; Copyright 2011 John Wiley and Sons.

The highest off-resonant α_ME_ of 52 V/cm Oe was realized in PMN-PT single-crystal fiber laminated with six layers of magnetostrictive Metglas at 1 kHz in multiple L-L mode [192], as shown in Figure 2a,b. The multiple L-L acoustic mode leads to an alternative expansion and compression of the lattice in the piezoelectric layer, as shown in Figure 2c. The multi-push–pull modality in the ME composite was configured by a pair of Kapton interdigital (ID) electrodes, where the alternating electric field streamline is shown in Figure 2d. The final Metglas/ID electrodes/PMN-PT ME sensor is shown in Figure 2e. An extremely low equivalent magnetic noise of 5.1 pT/Hz^1/2^ @ 1 Hz was realized [64]. A similar performance of 8.2 pT/Hz^1/2^ @ 1 Hz was also realized in a 4 × 4 Metglas/Pb (Zr, Ti) O_3_ magnetoelectric (ME) sensor array unit in an open environment [188]. By magnetic frequency conversion (MFC), low limit of detections of 20 pT @1 Hz, 150 pT @ 0.1 Hz, and 200 pT @ 0.01 Hz were demonstrated owing to up-conversion of the frequency band near EMR for high sensitivity and rejection of low-frequency noise [204]. The details of this active detection scheme are elaborated upon in Section 3.1.2.

Despite the success of the direct detection scheme in the realization of pT-level magnetic field sensing, a DC bias field typically needs to be applied by permanent magnets or solenoids on the ends of bulk ME sensors to realize maximum ME coupling and best sensitivity. The use of magnets makes the device bulky and adds additional magnetic noise sources and electromagnetic interference [17]. The mutual interference between each ME sensor also makes the integration of the sensor array challenging [205]. To realize non-zero ME coupling at a zero-bias field, self-biased ME composites (SMECs) have been developed in the last two decades. The self-biased ME effect was first discovered in co-fired LSMO-PZT laminates in 2002 [206], followed by another discovery in Fe–PZT–Fe in 2005 [207]. Existing self-biased ME composites can be classified as functionally graded FM-based SMECs [207,208,209,210,211], exchange bias-mediated SMECs [200,212], magnetostriction hysteresis-based SMECs [208,213,214], built-in stress-mediated SMECs [2,206,215,216], and non-linear SMECs [217,218,219]. The detailed development of SMECs can be found in [179].

#### 3.1.2. Thin-Film ME Sensor

Compared to bulk ME sensors suffering from piezoelectric leakage and non-ideal glued mediated mechanical coupling, thin-film ME sensors realize atomic interfacial bonding between a magnetostrictive layer and a piezoelectric layer. The thin-film ME sensors show an ultra-compact size from mm to μm and show great potential for high spatial resolution magnetic sensing. The EMR of the compact thin-film ME sensors is in the kHz or MHz frequency range, which is far beyond the frequency band (0.01 Hz~1 kHz) of the magnetic signal to be detected. The ultra-low sensitivity at off-resonance mode and high 1/f noise levels make the direct passive detection scheme impractical. Indirect detection schemes have been developed for the operation of thin-film ME sensors, including magnetic frequency conversion (MFC), electrical frequency conversion (EFC), and the delta-E effect [190].

Magnetic frequency conversion relies on the quadratic magnetostriction effect versus a bias field and converts the low-frequency magnetic signal to the sidebands near EMR to ensure high magnetic sensitivity for practical magnetic sensing [217,218]. When a driving modulation AC field at a high-frequency *f*_mod_ near EMR is applied to the ME sensors together with the magnetic signal at *f*_sig_, the output of the ME sensors includes the product of the two signals due to quadratic magnetostriction. The sensor output frequency spectrum contains *f*_mod_ − *f*_sig_, *f*_mod_, and *f*_mod_ + *f*_sig_, where the low-frequency noise is highly suppressed. However, the modulation magnetic field is typically generated by a modulation coil, which introduces additional high-frequency noise and high power consumption for driving it. The coil also limits the size miniaturization of the ME sensor system. To overcome these challenges, electrical frequency conversion has been developed, which exerts modulated voltage on the piezoelectric layer. The modulated voltage leads to periodic stress on the magnetostrictive layer and induces additional uniaxial magnetic anisotropy, affecting magnetization dynamics. Although a modulation coil is not required, the output electronics must have a large dynamic range to stay outside the saturation region and provide a strong modulation voltage for detecting very weak magnetic signals [11,17]. This can restrict the sensor’s ability to detect both very small and very large magnetic field variations. Electrical frequency conversion introduces noise from electronic components, like mixers, oscillators, and amplifiers [220]. These components, especially in low-power systems, are prone to phase noise and thermal noise, which degrade the sensor’s signal-to-noise ratio (SNR). As a result, the converted signal is often less pure and contaminated, making it harder to detect weak magnetic fields. The equivalent magnetic noise performance of the EFC-based ME sensor is limited to several nT/Hz^1/2^ [11].

For both MFC and EFC, the frequency up-conversion boosts the noise level while amplifying the magnetic signal. The optimal driving field or voltage is obtained by the balance between increasing the signal strength and maintaining a manageable noise level. The frequency up-conversion method often requires more complex circuitry and additional power to maintain the modulation and detection processes. This can limit its practical applications in low-power, portable devices or in scenarios where simplicity and energy efficiency are critical. While the resonance frequency is selected to enhance the detection capabilities, it also limits the sensor’s performance in a broad frequency range. Tuning to a specific frequency can make the sensor less effective for detecting magnetic fields that fall outside of this range. The output signal distortion and the introduction of additional spurious signals due to non-linear mixing are also problems that need to be addressed.

The delta-E effect is an alternative method that utilizes a resonance frequency shift in the ME resonator owing to the Young’s modulus change of the magnetostrictive material under a magnetic field excitation. This method can effectively enhance sensitivity, since the resonance frequency shift is more sensitive to external magnetic field signal change [221]. The dynamic range of ME sensors can also be enhanced because the sensors can operate on multiple mechanical resonance modes [221]. For instance, by applying different adapted electrode designs on a FeCoSiB (2 μm)/poly-Si (50 μm)/AlN cantilever beam (2 μm), the first and second transversal bending modes could be excited [221]. The sensitivity was boosted by a factor of six, and the dynamic range of the sensor output was reduced by 16 dB, which significantly eases the bandwidth requirements of readout electronics. The wide bandwidth of 100 Hz and an equivalent magnetic noise of 100 pT/Hz^1/2^ make the delta-E effect sensors promising for low-frequency magnetic field sensing [221].

Depending on the structure, size, and excitation configurations of the ME sensors, different bulk acoustic wave modes can be established for magnetic sensing under certain indirect detection schemes. Based on the structure of the ME sensors, they can be divided into cantilever-based ME sensors, nanoplate resonator (NPR) ME sensors, nanoplate resonator (NPR) array ME sensors, etc. The excited bulk acoustic wave modes and sensor performances were discussed.

##### Cantilever-Based ME Sensor

**Figure 3:**
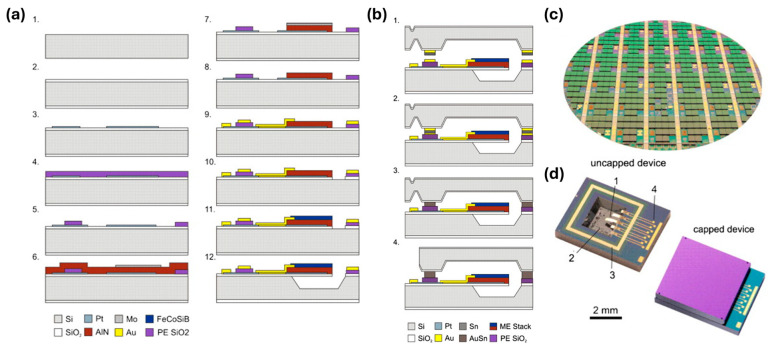
(**a**) Process flow of cantilever-based ME sensors. (**b**) Bonding process flow of capped wafer to effectively improve the mechanical quality factor and reduce the equivalent magnetic noise. (**c**) Photographs of ME sensor die on wafer (**d**) cantilever-based ME sensor with (1) ME cantilever, (2) etch groove, (3) bond frame, and (4) bond pads. Reprinted with permission from Ref. [222]. Copyright Elsevier 2013.

The cantilever-based ME sensors are typically composed of a surface-micromachined magnetostrictive layer and a piezoelectric layer on both sides of a poly-Si or thermal oxide Si substrate. The fabrication of cantilever-based magnetoelectric (ME) sensors involves a multi-step process that integrates both piezoelectric and magnetostrictive layers on the substrates to produce highly sensitive, miniaturized sensors [222], as shown in Figure 3a. The process typically initiates with the deposition of Ti/Pt layers to form the bottom electrode, followed by PECVD deposition of SiO_2_ for electrical isolation. Subsequently, piezoelectric layers, such as AlN, are deposited and structured using Mo hard masks and selective etching techniques. Following this, a Cr/Au layer is added to create conduction lines and electrical bond pads. For the magnetostrictive layer, materials like (Fe_90_Co_10_)_78_Si_12_B_10_ are deposited with Ta seed and capping layers, ensuring both adhesion and enhanced magnetic response. Finally, a selective silicon etching using XeF_2_ and TMAH releases the cantilever, resulting in a high-aspect-ratio structure that responds to magnetic fields with a measurable piezoelectric output.

The first thin-film cantilever beam AlN/Si/FeCoSiB ME sensor was fabricated by Jahn et al. in 2011 [223], where a 20 mm × 3 mm silicon cantilever was covered with a 0.3 μm-thick molybdenum bottom electrode and a 1.8 μm-thick piezoelectric AlN on top. A 1.75 μm-thick magnetostrictive FeCoSiB top electrode with an area of 1.6 × 10^−5^ m^2^ acted as a third layer. A 7 mm broad trench was etched to reduce the mechanical resonance frequency to 330 Hz. The cantilever sensor was clamped onto an epoxy block. The maximum ME coefficient of 1200 V/cm Oe was realized at a 6 Oe bias field. The noise of the ME sensor system mainly comes from the intrinsic thermal noise of the ME sensor and the extrinsic noise of voltage and charge amplifiers. An equivalent magnetic noise of 5.4 pT/Hz^1/2^ was obtained at 330 Hz. Compared to the calculated noise spectrum, additional noise was found to come from magnetic coupling to ambient noise sources, such as transformers, switching power supplies, electric motors, and external mechanical vibrations at its bending resonance. A similar surface micromachined cantilever beam with a stack composed of SiO_2_/Ti/Pt/AlN/Cr/FeCoSiB was fabricated on a 150 mm Si (1 0 0) wafer [224], realizing a giant ME coefficient α_ME_ = 1000 (V m^−1^)/(A m^−1^) in resonance at 2.4 kHz. The resulting static ME coefficient is α_ME_ = 14 (V m^−1^)/(A m^−1^). In resonance operation, a sensitivity of 780 V/T and noise levels as low as 100 pT/Hz^1/2^ have been reached. The wafer bonding and encapsulation can effectively improve the mechanical quality factor and reduce the equivalent magnetic noise from 100 pT/Hz^1/2^ [224] for the non-encapsulated device to 27 pT/Hz^1/2^ [222]. Since commonly used glass frit and anodic bonding require temperatures beyond the 300 °C threshold for glass to crystallize and change properties, a transient liquid phase (TLP) bonding process was specially developed with a bonding temperature of 260 °C [225]. The bonding process is shown in Figure 3b. The cap wafer and sensor wafer are carefully aligned and loaded into a wafer bonder with a vacuum pressure of 5×10^−5^ mbar. By heating to 260 °C for 10 min, the melt and solid-liquid interdiffusion of the thin interlayer led to isothermal solidification, forming a robust bond between the wafers. The wafer-level packaged (WLP) magnetoelectric MEMS sensors after dicing were encapsulated [224], as shown in Figure 3c,d. In addition to encapsulation, PZT films with interdigital transducer electrodes were used as a piezoelectric phase for the Si cantilever ME sensors based on FeCoSiB films on the backside [186]. The limit of detection was improved to 2.6 pT/Hz^1/2^ at the mechanical resonance of ~975 Hz [186], which is only about 1/3 of the AlN-based ME sensor [147].

To improve magnetic sensitivity, a 1000 μm × 200 μm poly-silicon (5 μm)/Sc-doped AlN (Al_0.73_Sc_0.27_N, 1 μm)/FeCoSiB (2 μm) cantilever ME sensor was developed in 2020 [226]. The 1.38-fold increase in the piezoelectric coefficient and the relative permittivity ratio between AlScN and AlN [93] nearly doubles the magnetoelectric (ME) voltage coefficient, from 734 ± 38 V/cm/Oe for AlN ME sensors to 1334 ± 84 V/cm/Oe for AlScN ME sensors [226]. This enhancement is especially beneficial for applications that do not operate at the noise limit, such as current sensors, where higher sensitivity can reduce demands on the electronic system, thus lowering production costs [226]. However, the in-resonance detection limit remains nearly unchanged at 60 ± 2 pT/Hz^1/2^ for AlScN-based sensors compared to 62 ± 2 pT/Hz^1/2^ for AlN-based sensors, as the increased piezoelectric response boosts both thermomechanical noise and voltage output. Outside of resonance, however, the detection limit for AlScN-based devices shows a 1.85-fold improvement [226]. In addition to improving the properties of the piezoelectric layer, the magnetostrictive layer was improved by direct deposition on the smooth surface of the Si/SiO_2_ cantilever instead of on an AlN film [185]. The inverse bilayer ME sensor was enabled by reactive sputtering of an Al target powered by a 250 kHz pulsing unit in a N_2_ environment without intentional substrate heating. The room temperature deposition maintains the amorphous nature and superior magnetic properties of FeCoSiB, which cannot be realized in the process flow with high-temperature AlN deposition. A high-quality factor of 310 and ME coefficient of 5 kV/cm/Oe was realized at an EMR of 867.8 Hz, leading to the lowest equivalent magnetic noise of ~400 fT/Hz^1/2^.

Starting from 2011, MFC has been used in both bulk ME sensors [227] and thin-film ME sensors [147]. In 2012, Jahn et al. utilized the MFC to realize a 1000-times sensitivity enhancement from unmodulated 1 μT/Hz^1/2^ @ 1 Hz to modulated 1 nT/Hz^1/2^@ 1 Hz. However, this equivalent magnetic noise is much worse than 7.1 pT/Hz^1/2^ in direct measurements due to the undesired additional noise under modulation from Barkhausen noise originating from magnetization jumps of pinned domain walls near defects. To reduce the magnetic noise, exchange bias stack 3 × (5 nm Ta/3 nm Cu/8 nm Mn-Ir/FeCoSiB) was first developed in 2013, which can realize a stable single domain state, indicating a well-defined magnetization reversal by coherent magnetization rotation for the FeCoSiB layer up to 100 nm with a ME coefficient of ~340 V/cm/Oe [212,228,229,230,231]. The structure of the stack was not altered up to 250 °C [229]. In 2015, the Ta 5 nm/Cu 3 nm/MnIr 8 nm/FeCoSiB 200 nm exchanged bias stack-based cantilever ME sensor was first demonstrated under MFC. Limits of detection of 180 pT/Hz^1/2^ @ 10 Hz and 300 pT/Hz^1/2^ @ 1 Hz using MFC were realized, which is 3 orders of magnitude better than 10 nT/Hz^1/2^ @10 Hz for a ~3 μm thick non-biased stack [230]. Using this exchange bias multilayer stack, a generalized numerical model was proposed to examine magnetic excess noise [232], which is the main noise source that arises with a pumping signal. It was found that the magnetic excess noise can be reduced by a DC-biased pump signal, thereby enhancing the signal-to-noise ratio (SNR) and improving LOD.

**Figure 4:**
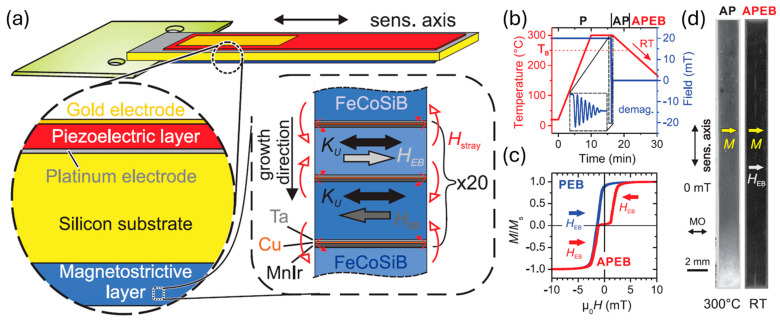
Magnetically modulated cantilever ME sensor with antiparallel exchange bias (APEB) stack. (**a**) The sketch illustrates the ME cantilever design with the magnetic field sensing axis indicated. A cross-sectional view highlights the ME sensor’s structure, showing the Si substrate, a piezoelectric AlN layer, a Pt and Au electrode, and the magnetostrictive layer. The magnetostrictive layer consists of a repeated multilayer structure with Ta/Cu seed layers, an antiferromagnetic MnIr layer, and a magnetostrictive FeCoSiB phase. This structure includes a 20× repeated two-layer configuration. Key magnetic parameters are indicated, including the uniaxial anisotropy axis K_u_, the exchange bias field H_eb_, and the magnetic stray field distribution H_stray_. (**b**) A diagram shows the temporal application of temperature and magnetic field during the annealing process. (**c**) An inductive measurement shows the magnetization loop along the alternating EB axis, with a magnetization loop from a parallel exchange-biased (PEB) sample included for comparison. (**d**) Magnetic domain (MD) structures at 300 °C after demagnetizing the sensor and achieving a stabilized magnetization state at room temperature (RT) for the APEB sensor. The alignment of magnetization M, the EB field H_eb_ in the top layer, the sensor’s magnetic field sensing axis, and the magneto-optical sensitivity (MO) axis are depicted. Reprinted with permission from Ref. [181]. Copyright 2019 AIP Publishing.

**Figure 5:**
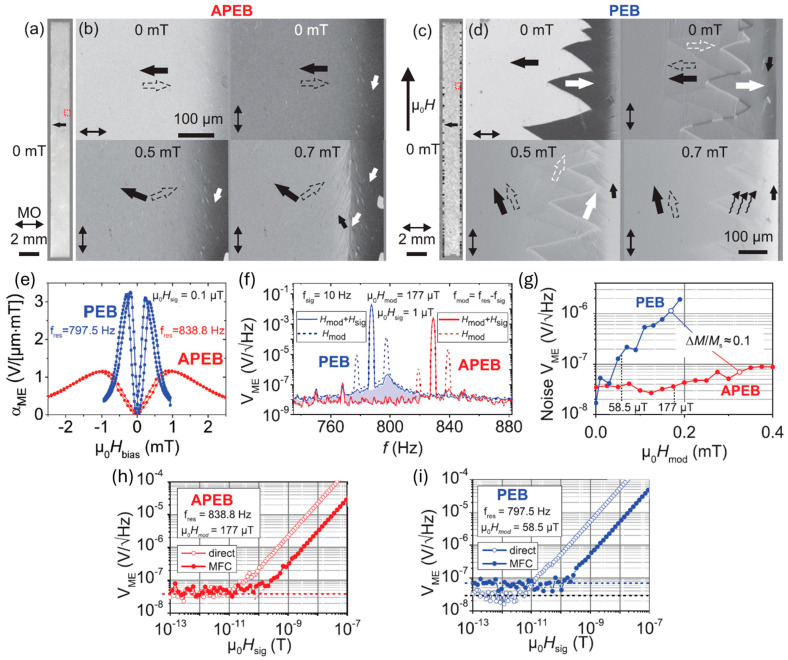
Magnetic domain behavior and noise performance of magnetically modulated cantilever ME sensor with antiparallel exchange bias (APEB) stack and PEB stack. (**a**) A full-sensor view of the APEB’s magnetic domain structure, with magnetization directions marked by arrows. (**c**) A full-sensor view for the PEB sensor, highlighting differences in magnetic domain patterns compared to APEB. (**b**,**d**) Provide high-resolution domain images of specific regions at the right cantilever edge (indicated by red dashed boxes in (**a**,**c**)) for both APEB and PEB, respectively, illustrating domain behavior with and without an applied magnetic field. Dashed arrows denote the magnetization alignment in the second, non-visible FeCoSiB layer. (**e**) The magnetoelectric (ME) coefficient α_ME_ changes with bias field H_bias_ at the mechanical resonance frequency f_res_. (**f**) Frequency spectra of voltage noise density V_ME_ for both APEB and PEB sensors under the same modulation H_mod_ and signal fields H_sig_. (**g**) Voltage noise dependency on H_mod_, highlighting the sensitivity to modulation field strength. (**h**) and (**i**) show linearity plots of f_res_ and magnetic frequency conversion (MFC) mode for a ME sensor with APEB and PEB phases, respectively. Noise floors and optimal H_bias_ and H_mod_ values for resonance and MFC modes are indicated, showing comparative sensor performance across configurations. Reproduced with permission from Ref. [181]. Copyright 2019 AIP Publishing.

In the parallel exchange-biased (PEB) antiferromagnetic/ferromagnetic (AF/FM) stack demonstrated in 2012, limited magnetic domain control can only guarantee a single domain state for FeCoSiB thickness below 100 nm [212,228]. Due to the demagnetizing field, magnetic domains still form in a thick FeCoSiB layer, which limits the maximum achievable sensitivity and results in a poor LOD. In 2019, a cantilever ME sensor based on an antiparallel exchange bias (APEB) stack was developed, which can lead to single magnetic domain state formation without magnetic layer thickness restriction. The sensor structure and magnetic stack are shown in Figure 4a [181]. The Ta (25 nm)/Pt (150 nm)/AlN (2000 nm)/Cr (5 nm)/Au (100 nm) was sputtered on top of double-sided polished 350 μm-thick thermally oxidized Si, while the APEB stack was deposited on the bottom. The stack comprises 20 × [Ta(5 nm)/Cu(3 nm)/Mn_3_Ir(8 nm)/(Fe_90_Co_10_)_78_Si_12_B_10_(90 nm)/Ta(5 nm)/Cu(3 nm)/Mn_3_Ir(8 nm)/(Fe_90_Co_10_)_78_Si_12_B_10_ (110 nm)]. The imbalance of the adjacent FeCoSiB layer thickness leads to the difference in Zeemann energy and switching field of the two adjacent layers, easing the antiparallel alignment of magnetization and facilitating an antiparallel magnetostatic coupling of the two layers. The stack was first annealed at 300 °C with a 200 Oe field along the short axis of the cantilever to form PEB, as shown in Figure 4b. A decaying sinusoidal AC magnetic field followed by a linear cooling process was used to degauss the stack and transit PEB to APEB magnetization due to the magnetostatic interaction. The dominant bipolar APEB state was confirmed by the hysteresis in Figure 4c and field-dependent MOKE images in Figure 4d. The domain rotations in the APEB and PEB stack are shown in Figure 5a–d. In APEB, a single domain state was observed on the top surface, while magnetic domains were observed on the edge in PEB due to the demagnetizing effect. Applying a field along the long axis leads to a coherent magnetization rotation in the APEB stack without domain wall activity visible. However, in the PEB stack, immediate domain wall activity and compensated Néel wall structures are clearly visible. The invisible weak domain wall activity makes the APEB sensor show a significantly lower flat noise level than PEB (Figure 5f), even though the APEB stack has an operational frequency shift and a lower maximum magnetic sensitivity in the absence of the demagnetizing effect (Figure 5e). Under MFC, the noise performance of APEB is improved by more than one order of magnitude relative to the PEB sensor. In contrast to the PEB sensor, where the noise level increases steadily with a rising *H*_mod_, the noise level of the APEB sensor remains stable (Figure 5g). This means the noise level of the APEB sensor is dictated solely by thermomechanical noise [178,223,233] independent of the detection scheme, while the PEB sensor still suffers from the dominant magnetic noise. The rejection of magnetic noise in the APEB sensor makes it have a LOD as low as 60 pT/Hz^1/2^ at 10 Hz, which is half that of the PEB sensor.

In addition to magnetic excess noise, acoustic and vibrational noise is inherent in any acoustic mode and has a spectrum in the low-frequency region up to several kHz. Even though it can be suppressed by the shielded chamber during tests, vibrational noise cancellation is still necessary for practical applications. In 2016, a tuning fork consisting of two identical cantilever ME sensors on top and at the bottom of a mounting block was proposed to strongly reduce the acoustic and vibrational noise by canceling out vibrational couplings by means of analog signal processing [187,234]. The sensors, which utilize interdigital (ID) electrodes, comprise a multilayer stack of Ta (5 nm)/FeCoSiB (4 µm)/Si (300 µm)/ZrO_2_ (300 nm)/PZT (2 µm)/Cr (5 nm)/Au (100 nm) and work on the first bending mode at 958 Hz. A limit of detection of ~500 fT/Hz^1/2^ was realized for the tuning fork without an artificially added wideband piezoelectric loudspeaker, which is one order of magnitude better than 5 pT/Hz^1/2^ for a single cantilever ME sensor. With the acoustic-disturbing source, the single ME sensor shows two orders of voltage noise level and LOD decrease, while the turning fork ME sensor only degrades by a factor of four. To address the wide dynamic range requirement due to the large carrier signal leak to output under unbalanced magnetostriction characteristics, a carrier suppression technique was recommended [235]. This technique successfully reduced the carrier signal leakage by 60 dB and avoided oversteering the readout electronics, reducing the analog-to-digital conversion challenges [235].

**Figure 6:**
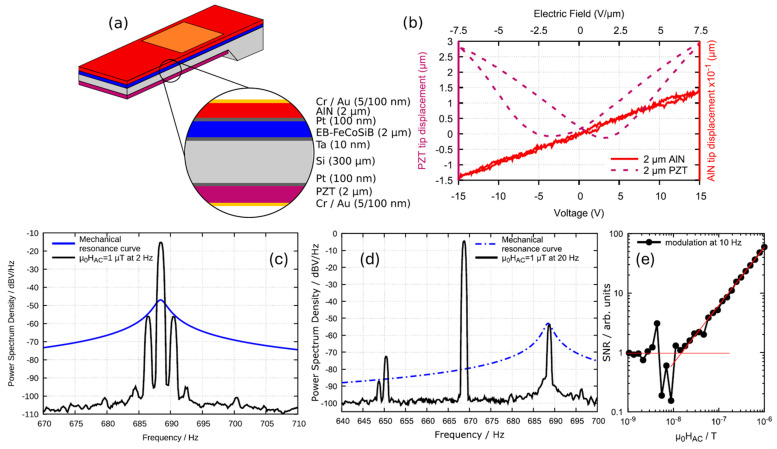
Electrically modulated ME sensors with AlN layer for voltage output. (**a**) The schematic shows a magnetoelectric (ME) composite sample with three active layers: an exchange-biased FeCoSiB layer serving as the magnetostrictive phase, an AlN layer as the linear piezoelectric phase for readout, and an unpoled PZT layer as the nonlinear piezoelectric phase for excitation. (**b**) Displacement–voltage characteristic curve of the ME cantilever showing responses of both piezoelectric phases under a DC electric field. (**c**) The sensor output spectrum from the AlN layer when the PZT layer is excited at its mechanical resonance frequency of *f*_mod_ = 689 Hz. (**d**) Sensor output spectrum from the AlN layer with the carrier signal frequency *f*_res_ = *f*_mod_ = 669 Hz, applied at 20 Hz below the mechanical resonance. (**e**) A linearity test under a 10 Hz magnetic field demonstrates that the noise floor reaches approximately 10 nT/√Hz. Reproduced with permission from Ref. [11]. Copyright 2016 AIP Publishing.

Electrically modulated cantilever ME sensors were first demonstrated in 2016 by Hayes et al. [11] to mitigate the challenges of MFC in size and energy consumption, potential crosstalk in sensor arrays and external interfering stray fields. In this method, the low-frequency magnetic signal is shifted to match the mechanical resonance of the sensor, while the electric modulation frequency is set to either the sum or difference of the resonance and signal frequencies. The sensor consists of an exchange-biased magnetostrictive multilayer, a non-linear piezoelectric actuation layer, and a linear piezoelectric sensing layer, as shown in Figure 6a. The electric modulation field is applied to the unpoled PZT layer showing a hysteretic, quadratic displacement with respect to applied DC voltage and a non-linear piezoelectric coefficient for EFC, while the linear AlN layer is used to detect the ME voltage, owing to its low loss tangent and high piezoelectric coefficient [236], as shown in Figure 6b. The instantaneous slope of the PZT displacement curve determines the piezoelectric conversion coefficients, which are reflected in the magnetostrictive layer’s response to small magnetic fields. Consequently, the AlN output voltage carries a signal resulting from the commutation between the high-amplitude carrier signal and the low-amplitude magnetic signal, akin to magnetic frequency conversion. Using two distinct piezoelectric layers provides the added advantage of natural electrical isolation between the actuation voltage and the readout signal, enabling the potential for operation without the need for compensation mechanisms. The high piezomagnetic coefficient of the exchange biased layer at a zero-bias field removes the need to apply a DC bias field to operate at the maximum magnetic sensitivity point. EFC was realized by applying a modulated voltage signal at mechanical resonance (*f_res_* = *f*_mod_ = 689 Hz) on the PZT layer when the magnetic field signal (1 μT, *f*_AC_ = 2 Hz) was applied in parallel. The output spectrum of the voltage signal at the AlN layer shows the maximum carrier signal peak at *f_res_* from electromechanical coupling to the PZT layer and two sidebands at *f*_mod_ ± 2 Hz. A limit of detection of ~5 nT/Hz^1/2^ was obtained, as shown in Figure 6c. When the modulation frequency is set to *f_res_ − f_AC_* = 669 Hz, the LOD slightly increases to ~10 nT/Hz^1/2^ due to thermal noise [237], as shown in Figure 6d,e. The advantage of EFC is that it allows the sensor to function at substantially higher resonance frequencies, which are difficult to reach with magnetic excitation due to the high magnetic field amplitudes needed. Higher resonance frequencies are beneficial for achieving wider bandwidths and reducing cross-sensitivity to acoustic noise and vibrations. A limitation of this approach arises from the resonator’s high-quality factor, which confines resonance amplification to a narrow frequency range of typically only a few Hz.

**Figure 7:**
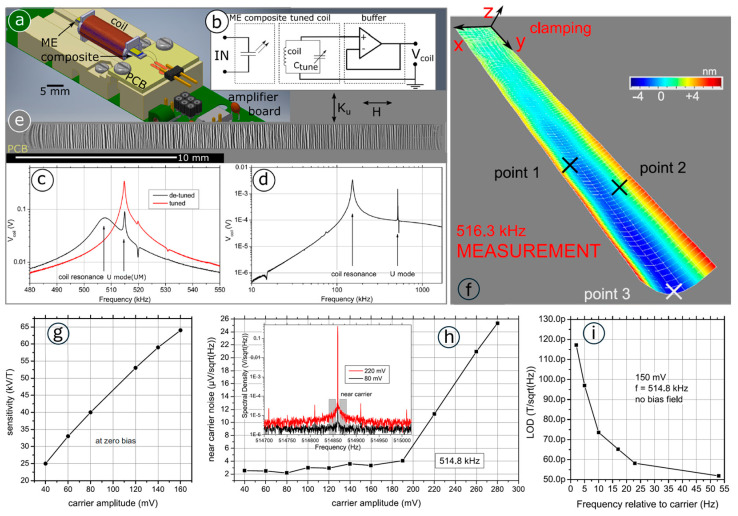
Electrically modulated U-mode ME sensors with a picking-up coil for voltage output. (**a**) Schematic setup: a composite, embedded in a pickup coil, is shown. The piezoelectric (PE) plate capacitor functions as the input, while the tuned pickup coil, coupled with an amplifier, provides the output signal. (**b**) Circuit representation: the ME composite is modeled as a radiative capacitor. The pickup coil generates a signal that is buffered by a low-noise, unity-gain buffer amplifier, enhancing signal integrity. (**c**) Frequency response analysis: the self-resonant frequency of the pickup coil (with quality factor Q∼150) and the mechanical resonance frequency (with quality factor Q∼1000, labeled as UM) are shown. Tuning the system maximizes voltage output at resonance, with de-tuning options also analyzed. (**d**) Wide frequency response: a broad response shows coil resonance effects and two distinct voltage peaks correspond to mechanical resonances of the system. (**e**) MOKE microscopy image: the full cantilever length is captured after magnetic field decay, showing magnetization along the thermally induced magnetic easy axis (K_u_). Applied magnetic fields (H) align with the hard axis, while the left end of the cantilever is fixed to the PCB. (**f**) Vibrometry measurements showing the U-mode at 514.8 kHz. (**g**) Sensitivity at zero field showing a linear increase with rising carrier voltage amplitude. (**h**) Voltage noise characteristics versus carrier amplitude. At low frequencies (<20 Hz), two regimes are identified based on carrier amplitude. Below 200 mV, noise increases only slightly, while above 200 mV, noise surges by nearly seven times for an additional 100 mV of excitation. Inset shows the noise spectra for 80 mV and 220 mV cases; the 220 mV excitation exhibits a noticeable pedestal and increased broadband noise. (**i**) LOD is assessed across different test frequencies, showing an exponential noise increase towards the carrier, which limits sensor performance. Reproduced from Ref. [10].

In addition to the AlN layer, the readout can also be performed by a pickup coil wound around the sensor [238,239,240]. In 2018, a mechanically decoupled pickup coil was used to detect the electrically modulated signal at quasi-DC frequencies from a 2 μm AlN/2 μm FeCoSiB cantilever [12]. The 15th flexural mode at 515.7 kHz and the 19th torsional mode (U mode) at 520.7 kHz were used to modulate the detected signal at 0.2 Hz, showing a LOD of 1.2 nT. Utilizing this U mode, a cantilevered mesoscopic ME structure was electrically excited [10], as shown in Figure 7a,b. The pickup coil senses the modulated magnetic field owing to the inverse ME effect under a modulated voltage on the piezoelectric layer, which was buffered by a low-noise, unity-gain buffer amplifier as the final voltage signal output. By tuning the capacitance, the voltage signal from the U-mode mechanical resonance can be boosted by about one order of magnitude, as shown in Figure 7c,d. As shown in Figure 7e, multiple magnetic domains form along the magnetic easy axis, leading to notable magnetic sensor due to the Bakhusen jump and degraded noise performance. Despite this, the U mode mechanical resonance (Figure 7f) exhibits a much sharper peak, with a quality factor (*Q*_UM_) near 1000, which is about an order of magnitude higher than typical flexural modes. In flexural modes, oscillation losses are largely due to air damping [241,242], particularly in long cantilevers whose Q factor rarely exceed a few hundred in ambient conditions. In contrast, the U mode benefits from inherently reduced air damping, as fewer air molecules are displaced, allowing it to achieve a higher Q factor and, therefore, superior resonance sharpness and efficiency. The U mode demonstrates the highest sensitivity to small magnetic fields, particularly under moderate excitation levels [13]. The sensitivity and voltage noise spectrum are shown in Figure 7g,h respectively. The sensitivity at zero field shows a linear increase with a rising carrier voltage. At low frequencies (<20 Hz), two regimes are identified based on carrier amplitude. Below 200 mV, the noise increases only slightly, while above 200 mV, the noise surges by nearly seven times for an additional 100 mV of excitation. As shown in Figure 7i, a magnetic LOD of 210 pT/Hz^1/2^ @ DC was realized. Further increasing the signal frequency leads to the improvement of the LOD from 70 pT/Hz^1/2^ @ 10 Hz to 50 pT/Hz^1/2^ @ 53 Hz.

**Figure 8:**
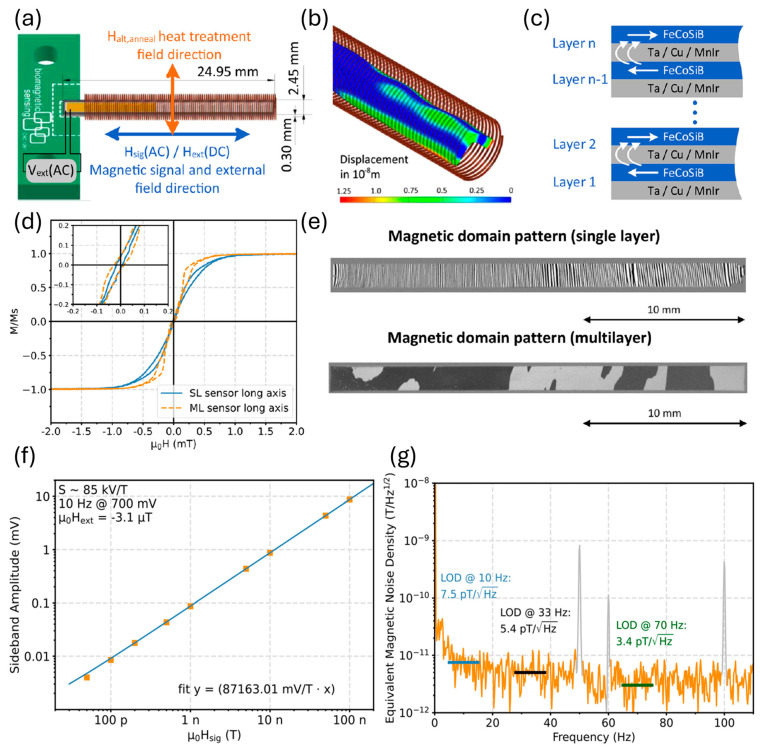
Low-noise inverse ME sensor based on electrically modulated U mode and exchange bias stack with a pickup coil. (**a**) Schematic of cantilever sensors in the inverse magnetoelectric configuration, featuring a simplified pickup coil covering the cantilever’s entire free length except where it connects to the circuit board. The measurement and bias field directions are shown by the blue arrow, while the orange arrow indicates the magnetic field direction applied during annealing. (**b**) Finite Element Method (FEM)-derived deflection of the cantilever in the U mode, depicted with amplified amplitude to illustrate bending. (**c**) Diagram of the advanced layer stack design for magnetic flux closure, showing Ta/Cu/MnIr layers, with each exchange-coupled to a FeCoSiB layer. The arrows represent potential magnetic flux closure and the preferred magnetization direction without cantilever excitation. (**d**) BH-Looper hysteresis curves for heat-treated single-layer (SL) and multi-layer (ML) samples (8 × 500 nm exchange bias layers) measured along the cantilever’s long axis, both with a total FeCoSiB thickness of 4 μm. (**e**) Magneto-optical Kerr micrographs of demagnetized domain patterns in SL and ML samples along the long axis, with sensitivity axis oriented along the cantilever’s short side. (**f**) Sideband amplitude response at several magnetic test signal amplitudes at 10 Hz, demonstrating extended linear behavior. (**g**) Equivalent magnetic noise density with limit of detection (LOD) calculated at 10 Hz (blue), 33 Hz (black), and 70 Hz (green), showing noise reduction and improved LOD at higher frequencies with a constant sensitivity of 85 kV/T across test fields from 10 to 70 Hz. Reproduced from Ref. [13].

In 2024, a low noise exchange bias stack was first used to reduce magnetic noise in an EFC U-mode cantilever ME sensor with a pick-up coil for the voltage output [13], as shown in Figure 8a,b. The advanced layer stack design was performed for magnetic flux closure, where each Ta/Cu/MnIr layer was exchange-coupled to a FeCoSiB layer, as shown in Figure 8c. The B-H loop is shown in Figure 8d. Around zero magnetic field, a small hysteresis opening becomes visible due to individual local switching of magnetic domains at edges or individual switching of domains in the different layers with areas of different effective anisotropy in the multilayer. The observed nonlinear magnetization curve constrains the dynamic range of the sensor. In the multilayer exchange bias stack, no closure domains develop, indicating a dominant stray field coupling between the magnetic layers. This potentially leads to a reduction of magnetic noise by reducing the domain wall density, as shown in Figure 8e. Furthermore, the exchange bias pins the magnetic domain walls and, thus, reduces or eliminates domain wall mobility. As shown in Figure 8f, the sideband voltage amplitude increases linearly with the magnetic test signal from 50 pT to 100 nT, where a constant sensitivity of 85 kV/T across the test fields was demonstrated from 10 to 70 Hz. The detection limits are improved by an order of magnitude to less than 8 pT/Hz^1/2^ at 10 Hz and 18 pT/Hz^1/2^ at DC, as shown in Figure 8g.

**Figure 9:**
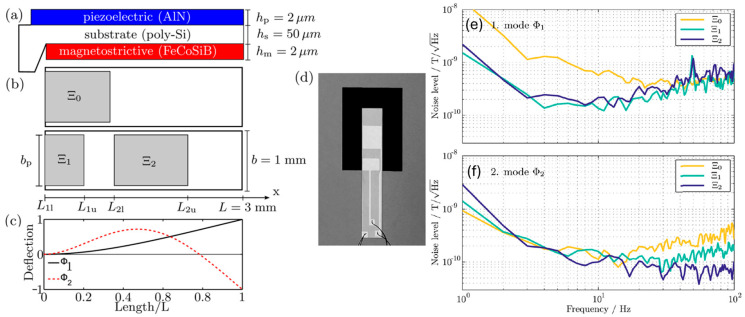
Multimode delta-E effect magnetic field sensors with adapted electrodes. (**a**) Schematic cross-section of the cantilever beam, (**b**) top view of the cantilever showing the various electrode designs, (**c**) calculated deflection of the cantilever in the first and second transverse bending modes according to Euler–Bernoulli beam theory, and (**d**) photograph of the sensing cantilever mounted within a silicon frame. Effective noise level for the (**e**) first and (**f**) second modes. Reproduced with permission from Ref. [221]. Copyright 2016 AIP Publishing.

In 2015, the delta-E effect was first used in a cantilever-based ME sensor [243]. The cantilever ME sensor is composed of 2 μm AlN and 2 μm FeCoSiB [243]. Operating in the first or second transversal bending mode at 7.6 kHz or 47.4 kHz, a limit of detection of 140 pT∙Hz^−0.5^ @ 20 Hz under a magnetic bias field and 1 nT∙Hz^−0.5^ @ 20 Hz without an external bias field was achieved [243]. Since the oscillation and vibration magnitude are small and linear, the interference between different modes can be rejected. In 2016, the adapted electrodes were developed to improve the sensitivity and reduce the dynamic range [221]. Different electrode designs of Ξ_0_, Ξ_1_ and Ξ_2_ were placed on top of the AlN (2 μm)/polySi (50 μm)/FeCoSiB (2 μm) cantilever ME sensor, as shown in Figure 9a,b. The first and second transverse bending modes can be excited, whose deflections are displayed in Figure 9c. The electrode design Ξ_0_ demonstrates high efficiency in the primary mode, Φ_1_, which is evident from its steep admittance change and significant phase shift, indicating robust energy coupling in this mode. However, its performance diminishes in the secondary mode, Φ_2_, where the response is less pronounced. In contrast, the adapted designs Ξ_1_ and Ξ_2_, which incorporate dedicated electrodes for each mode, provide enhanced operational efficiency across both modes. This design strategy ensures effective coupling for both Φ_1_ and Φ_2_, optimizing the sensor’s performance across a broader frequency range. The sensor was mounted on a silicon frame, as shown in Figure 9d. The dynamic range in the second mode shows a significant enhancement of 16 dB when transitioning from Ξ_0_ to Ξ_2_, indicating improved sensitivity and performance in this mode. The noise spectrums of different electrode designs at different modes are shown in Figure 9e,f. At low frequencies, noise levels increase due to resonance amplification, yet the second mode demonstrates superior performance above 20 Hz. This performance enhancement is especially pronounced with the optimized electrode design, Ξ_2_, which achieves a noise level below 100 pT/Hz^1/2^. This optimization makes Ξ_2_ highly effective for maintaining low noise levels in the second mode, enabling greater sensitivity and stability across a broader operational frequency range.

##### NPR ME Sensor

A 215 MHz self-biased ME MEMS magnetic sensor, based on a FeGaB/Al_2_O_3_ nano-plate resonator with precisely engineered inter-digital electrodes (IDEs), utilizes a high-quality ME MEMS resonator (quality factor, Q = 735) that is highly sensitive to DC magnetic fields near the electromechanical resonance (EMR), enabling an ultra-sensitive LOD of approximately 300 pT [180]. Upon applying a DC magnetic field to the ME heterostructure, the magnetostrictive strain in the FeGaB layer alters its Young’s modulus through the ΔE effect, resulting in shifts in EMR frequency and admittance of the MEMS ME resonator. This energy-efficient self-biased micro-scale MEMS magnetic sensor, based on a magnetostrictive/piezoelectric heterostructure, is compatible with CMOS fabrication and is promising for DC and low-frequency AC magnetic sensing applications.

**Figure 10:**
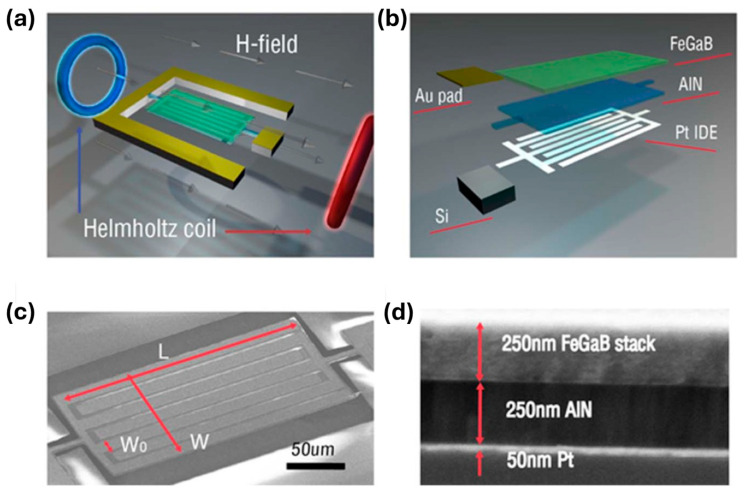
(**a**) Schematic and (**b**) layered structure of the ME sensor. Scanning electron microscopy images of the (**c**) fabricated MEMS ME sensor and the (**d**) cross-section. Reproduced from Ref. [180].

As illustrated in Figure 10a, a DC magnetic field was generated by Helmholtz coils and applied along the length direction. Figure 10b reveals the sensor’s layered structure. The AlN piezoelectric layer is flanked by FeGaB/Al_2_O_3_ magnetostrictive layers and Pt IDEs. The active sensing area (100 μm × 200 μm) is fully covered by IDEs and magnetostrictive material, as shown in the SEM image in Figure 10c. In this configuration, the extensional vibration mode is excited in the AlN film, with the EMR frequency given by:

$$f_{0}=\frac{1}{2W_{0}}\sqrt{\frac{E_{eq}}{\rho_{eq}}}$$

where *W*_0_ is the pitch width between IDEs, and *E*_eq_ and *r*_eq_ represent the equivalent Young’s modulus and density, calculated as $E_{eq}=\sum E_{i}v_{i}$ and $E_{eq}=\sum E_{i}v_{i}$, respectively, with *E_i_*, *r_i_*, and *v_i_* being the Young’s modulus, density, and volume ratio of each layer in the sensor. A high EMR frequency of 215 MHz was achieved by optimizing the IDE pitch width, which facilitated strong ME coupling due to a flat magnetostrictive piezoelectric interface. Furthermore, the removal of the underlying Si substrate minimized the substrate clamping effects, enhancing both ME coupling and sensitivity.

**Figure 11:**
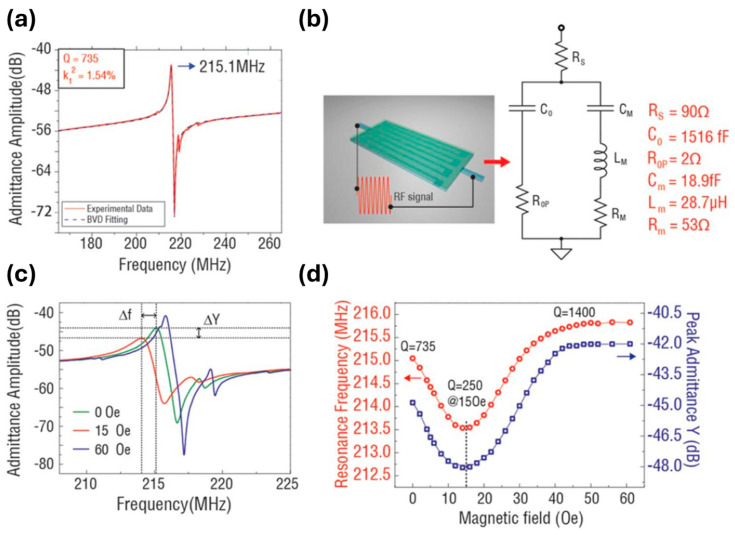
(**a**) MBVD model fitting for admittance curve of the ME sensor. (**b**) The equivalent MBVD circuit of the ME resonator. (**c**) Admittance curves of the ME resonator under different DC bias magnetic fields. (**d**) EMR frequency and peak admittance amplitude at the resonance frequency versus DC magnetic fields. Reproduced from Ref. [180].

Figure 11a depicts the measured admittance and its modified Butterworth–van Dyke (MBVD) model fit for the ME magnetic sensor, revealing an EMR frequency of 215 MHz. From the MBVD fit, a quality factor *Q*_m_ of 735 and an electromechanical coupling coefficient $k_{t}^{2}$ of 1.54% were extracted, with the $k_{t}^{2}$ being comparable to values for traditional AlN nano-plate resonators with similar electrode designs, while the *Q*_m_ is substantially higher than those typically observed in low-frequency ME magnetic sensors. Figure 11b presents the MBVD equivalent circuit for the AlN/FeGaB MEMS magnetic sensor, where *R*_s_, *C*_0_, and *R*_op_ represent the electrode resistance, ME resonator capacitance, and substrate parasitic resistance, respectively. Meanwhile, *C*_M_, *L*_M_, and *R*_M_ correspond to motional capacitance, inductance, and resistance [180].

In Figure 11c, the admittance response to various DC magnetic fields indicates strong ME coupling, as both the EMR frequency and admittance amplitude are affected by the ΔE effect. Figure 11d shows the resonance frequency and peak admittance at varying DC bias fields, where both initially decrease before increasing, correlating with the magnetic loss associated with domain wall activity. A minimum *Q*_m_ of 250 was reached at a transition field of 15 Oe.

##### NPR Array ME Sensor

**Figure 12:**
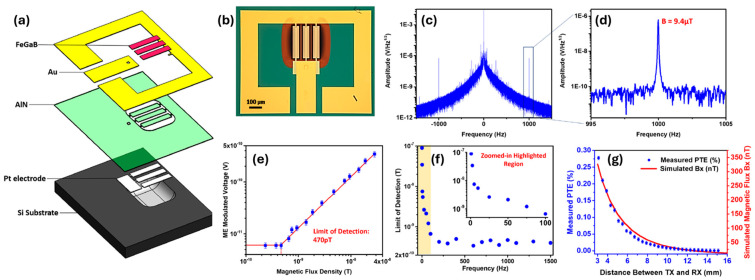
Self-biased NPR array dual-band ME sensor/antenna. (**a**) A 3D schematic of the ME sensor/antenna. (**b**) Optical image of a fabricated smart ME antenna. (**c**) Power spectrum of the reflected signal from the ME antenna after demodulation and lowpass filtering. (**d**) Zoom-in modulation signal at 1 kHz. (**e**) Modulated voltage as a function of modulated signal magnetic flux density, showing a 470 pT LOD. (**f**) LOD as a function of modulation signal frequency. (**g**) Power transfer efficiency as a function of distance between transmission and receiving antennas. Reproduced from Ref. [14].

Bio-implanted ME sensors are crucial for the contactless monitoring of nerve activity and disorders in magnetomyography [8,9]. In addition to sensing and recording neural signals with high spatial resolution and superior LOD in a wide range, the ME sensors need to efficiently harvest wireless energy to power themselves without batteries and transmit recorded neutral activity data to outside receivers. In 2017, an ultracompact NPR ME antenna was demonstrated, showing a LOD of 40 pT at 60.7 MHz [15]. However, the off-resonance operation at 1 MHz shows a poor LOD above 0.1 μT. Increasing the number of sensor elements is an effective way to boost the magnetic sensing capability. In 2021, a smart dual-band NPR array ME sensor/antenna was fabricated, as shown in Figure 12a,b [14]. The ME sensor consists of three parallel 500 nm AlN/500 nm FeGaB NPR ME resonators and has a width mode of 63.6 MHz. Each resonator shows an ultracompact size of 250 μm × 50 μm. The total size of the ME sensor is 250 μm × 174 μm. To boost the magnetic sensitivity at low frequencies, a MFC technique was applied by a 9.4 μT modulation AC magnetic field at 1 kHz. As shown in Figure 12, a sideband modulation signal of 6.2 × 10^−7^ V/Hz^1/2^ was detected at 63.301 MHz, and a 470 pT LOD @ 1 kHz was demonstrated in Figure 12e. The LOD as a function of the modulation frequency is shown in Figure 12e. Sub-pico-Tesla LOD was maintained from 100 Hz to 1000 Hz, while the LOD degrades fast below 100 Hz due to the dominant 1/f noise. This performance allows the sense to sensor the hundreds of pT-level neutral magnetic field signals at the pial surface [205]. Moreover, the dual-band ME sensor has a thickness mode of 2.53 GHz, which can be used for wireless energy transfer. The high wireless power transfer efficiency with an overwhelming figure of merit (FOM) over other micro-coils make the self-biased and passive dual-band ME sensors/ultra-high frequency (UHF) antennas promising for implantable biomedical magnetic sensing applications [14].

### 3.2. SAW Thin-Film ME Sensor

There are many SAW-based chemical, temperature, and biosensors available in the literature. In the current review, only SAW-based sensors that have both the piezoelectric and magnetostrictive layers are included. There are two types of SAW-based sensor concepts available, either a delay line [244] or a resonator [245]-type approach. Most of the demonstrated sensors work with the delay line configuration.

Such sensors consist of interdigital transducers (IDTs), typically made from Cr/Au, Al, or Ti/Au materials, on top of a piezoelectric layer/substrate (quartz, LiNbO_3_, and LiTaO_3_) and a magnetostrictive layer (for sensing). The basic physics behind the operation of SAW-based magnetic field sensors is the delta-E effect [21]. An electric signal on the IDTs produces an acoustic wave at one end of the sensor, and the acoustic wave travels along the delay line. A highly responsive magnetostrictive layer is present between two delay lines. The magnetostrictive layer changes Young’s modulus (ΔE) or shear modulus (ΔG) when an external sensing magnetic field (H_sense_) is present. The change in modulus causes a change in the velocity with which the acoustic wave travels. Finally, at the other end of the device, the output IDTs convert this acoustic wave into electrical signals. The delta-E effect causes a phase shift of the acoustic waves, and this phase shift is a function of the H_sense_. The sensitivity of the sensor is determined by the phase shift that can be caused by a magnetic field change and is reported with the unit of ‘°/mT’.

Though the initial SAW-based magnetic sensors were demonstrated in 1987 [244], only a few investigations followed up. This could be due to the availability and advancement in MEMS, piezoelectric, and magnetostrictive materials. Many different studies have been published in the last decade with a focus on SAW-based magnetic field sensing. Here, we report a few important ones.

An experimental study showed an increased sensitivity at low magnetic fields when the magnetic layer was changed from Ni to CoFeB (which exhibits increased magnetostriction) along the delay line of the sensor [245]. Zhou et. al. realized a SAW-based magnetic sensor using 200 nm TbCo_2_/FeCo on top of a LiNbO_3_ substrate. They use a Rayleigh wave that travels through the delay line. They report a change in the velocity of the wave as a function of the magnetic field. The discussed theoretical model agreed well with the experimental results [246].

**Figure 13:**
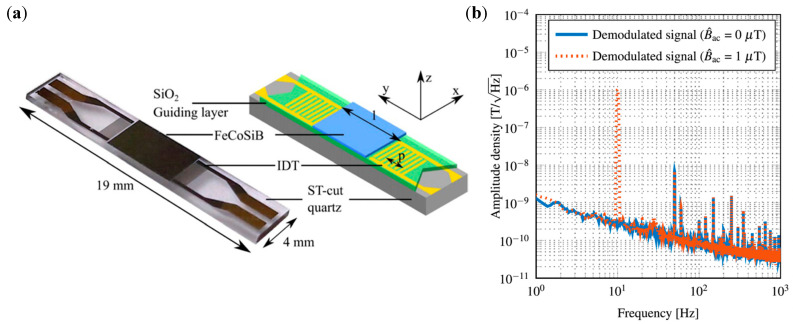
(**a**) Schematic of the wideband SAW-based magnetic sensor. The sensor is built on a ST–cut quartz substrate, with SiO_2_ guiding layer, and FeCoBSi as a sensing layer. (**b**) The image shows the frequency vs. amplitude density. The authors report a LOD of 80 pT/Hz^1/2^ at 100 Hz, a bandwidth of 50 kHz, and a dynamic range of 120 dB. Reprinted from Ref. [22].

Love waves, which are purely shear transverse-polarized waves, are widely utilized in magnetic field sensing, owing to ultra-sensitive response of the incorporated functional magnetic thin film to the target stimulus. A simulation for the structural sensitivity to the guiding layer (SiO_2_) thickness was investigated, and an optimal guiding layer thickness of 4.5 μm was determined and deposited on a ST-cut quartz substrate (Figure 13a) [22]. On top of this, a 200 nm FeCoSiB soft magnetic layer was deposited. The total sensitivity of the sensor depends on three different parameters, $S=S_{mag}\cdot S_{str}\cdot S_{geo}$, where $S_{mag}$ corresponds to the change in modulus for a change in magnetic field, limited by the magnetostriction of the material. The $S_{str}$ gives the change in the velocity for a change in modulus. It is limited by how well the acoustic wave is confined, and finally, $S_{geo}$ gives the change in the phase shift of the acoustic wave for a change in velocity. For the device fabricated the authors report a sensitivity of 504°/mT. The magnetic noise floor was plotted as a function of frequency and the LOD is frequency-dependent (Figure 13b). At 10 Hz a LOD of 250 pT/Hz^1/2^ and at 100 Hz a LOD of 80 pT/Hz^1/2^ have been demonstrated with a bandwidth of 50 kHz [22]. The total sensitivity of the SAW sensor can be enhanceed by increasing one of the three sensitivity parameters. The authors investigated magnetic films with multiple thicknesses of 25–400 nm [247]. As anticipated, the $S_{mag}$ increases with the thickness, but the insertion losses also increase at a higher thickness, which influences the LOD. Higher thickness leads to higher insertion losses, which in turn, increases noise and LOD [247]. They report that, for magnetic field sensing, it is best to keep the thickness between 50 and 250 nm.

**Figure 14:**
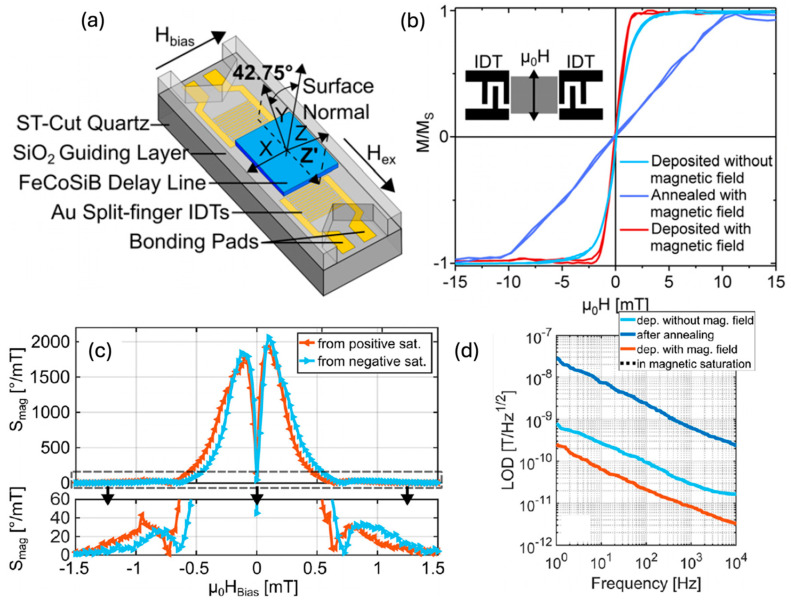
(**a**) The illustration of a Love-Wave-based magnetic sensor based on a ST–cut quartz substrate. (**b**) The magnetization loops of different configurations of stacks. Note that the one deposited under the in-situ field has low anisotropy (red) compared to the one with post annealing after film deposition (blue). (**c**) The measured sensitivity as a function of the applied DC bias field for the configuration deposited with in-situ magnetic field. (**d**) LOD as a function of frequency for different magnetic orientation configurations. The lowest LOD is from the device with the magnetic stack deposited with an in situ magnetic field. Reprinted with permission from Ref. [23]. Copyright 2020 AIP Publishing.

To further enhance the LOD of the SAW-based sensor, a well-defined magnetic film orientation is essential. In a 2020 study, the authors investigate different configurations to enhance the magnetic orientation 1) by depositing the magnetic film under an in-situ magnetic field (180 Oe) or 2) by depositing film without any field and conducting a magnetic field (1300 Oe) annealing at 270 °C for 30 min after film deposition (Figure 14a,b) [23]. They analyzed the magnetic properties, sensitivity, and LOD of the different configurations. They report the highest sensitivity of 2000°/mT (at 0.1 mT bias field) for the magnetic field SAW sensor with the in-situ field for magnetic film deposition (Figure 14c). For the annealed film after deposition, a sensitivity of 17°/mT (at 10 mT bias field) was obtained for the corresponding magnetic field SAW sensor. The reason for this difference is that, by depositing the film under an in-situ field, the precise orientation of the magnetic domains with low total anisotropy can be realized, which leads to high sensitivity. They also investigate the corresponding phase noise from which they report different LODs for different configurations. Once again, the configuration deposited with an in-situ field gives the best LOD of 70 pT/Hz^1/2^ at 10 Hz and 25 pT/Hz^1/2^ at 100 Hz (Figure 14d). It is reported that the noise at low frequency is dominated by the magnetic noise, and a multi-layer approach is further proposed to reduce the noise.

**Figure 15:**
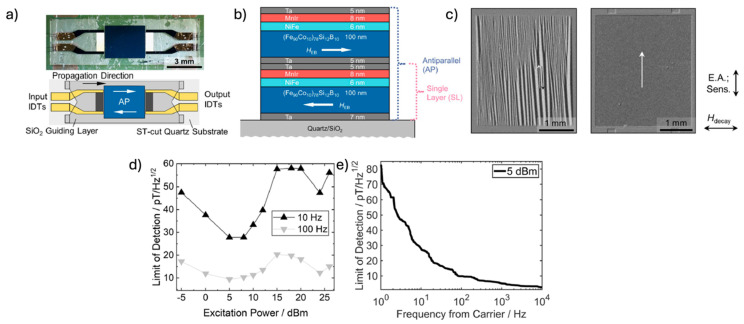
(**a**) The SAW sensor design consisting of a ST–cut quartz substrate, a SiO_2_ guiding layer and an anti-parallel exchange biased magnetostrictive layer (for sensing). (**b**) The layer stack of the magnetostrictive layer; it consists of two layers (Ta/FeCoSiB/NiFe/MnIr/Ta) that are antiparallel to each other. The NiFe/MnIr induces the exchange bias in the FeCoSiB layers of. (**c**) The magneto-optical Kerr effect microscope images from the top on a single FeCoSiB layer and a two-layer anti-parallel exchange bias stack. The image clearly shows the significant reduction in the domain wall density. (**d**) LOD plotted as a function of excitation power at 10 Hz and 100 Hz. Note that the LOD has the lowest value at 5 dBm for both frequencies. (**e**) Frequency spectrum of LOD with 5 dBm power. The LOD decreases as frequency increases, and an impressive LOD below 5 pT/Hz^1/2^ is achieved at 1 kHz. Reprinted from Ref. [25].

A 2023 study from Schell et al. showed an exchange bias-based SAW magnetic field sensor (Figure 15a) [25] to reduce the previously discussed magnetic noise at a low frequency. In the study, an anti-parallel alignment of an exchange-biased magnetostrictive layer is investigated on a ST–cut quartz substrate. The exchange bias stack with Ta (5 nm)/FeCoSiB (100 nm)/NiFe (6 nm)/MnIr (8 nm)/Ta (5 nm) layers was used to reduce the fluctuations that are caused by magnetic domain wall motion. A X-Ray Diffraction (XRD) pattern shows that the MnIr has a strong (111) texture, which is essential to achieve strong exchange bias. The (111) texture is realized by using a 6 nm NiFe seed layer. The anti-parallel alignment of the magnetic layer was obtained by depositing the film under an in-situ magnetic field. After the first layer deposition, the sample was rotated by 180°, and the second layer was deposited (Figure 15b). Such anti-parallel exchange-biased magnetic configuration showed a reduced magnetic domain wall density (Figure 15c) compared to a single layer, with the top of the magnetic layer showing a single domain state over the complete film. The sensor was characterized at different powers (0, 10, 15, and 24 dBm). Such anti-parallel exchange bias stacks lead to an increased sensitivity of up to ~2000°/mT from 0 to 15 dBm and achieved the highest sensitivity of 3250°/mT at 24 dBm. At a higher excitation power, the increase in sensitivity also increases the insertion losses, which further reduces the LOD. The LOD as a function of the excitation power is shown in Figure 15d for different excitation powers. For both 10 Hz and 100 Hz of the carrier frequency, the LOD first decreases and has a minimum between 5 and 10 dBm and then increases. The authors report a LOD of 28 pT/Hz^1/2^ at 10 Hz and 10 pT/Hz^1/2^ at 100 Hz (Figure 15e) at 5dBm, which is a factor of 2.5 lower compared to the single-layer stack. However, they point out that the mechanism of the influence of the excitation power on the phase noise needs further investigations for better understanding.

The efforts above focus on SAW-based devices based on quartz substrates. A CMOS-compatible SAW-based magnetic field sensor was proposed by Meyer et al. [24]. The sensor used magnetron-sputtered AlScN as a piezoelectric material, showing a LOD of 72 pT/Hz^1/2^ above 10 kHz. Even though the LOD is higher than the SAW sensors based on quartz, the CMOS compatibility could make such a device more practical in industrial applications.

A multifunctional sensor that is operated both in Rayleigh and Love wave mode was demonstrated by Yang et al. The sensor consists of a ZnO and SiO_2_ guiding layer on top of a quartz substrate. The ZnO is used here for insulating and impedance-matching purposes. The Rayleigh mode, on the other hand, is sensitive to temperature change, with a sensitivity of −37.9 ppm/°C. The Love wave is insensitive to the temperature difference. The authors use the Love wave mode for the magnetic field sensing, which is different from the previous approaches. Here, the authors measure the shift in the resonant frequency caused by the sensing magnetic field. They demonstrate a sensitivity of −170.4 kHz/mT for magnetic field sensing. A wireless sensor read-out system for magnetic field measurement is also investigated. It works as the sensing magnetic field detunes the SAW resonator, which can be remotely measured using a reader [248]. More recently, Hu et al. have performed FEM modeling and demonstrated a SAW-based self-biased magnetic field sensor. The authors report a strong dependence of resonant frequency on AC magnetic field when the in-plane uni-axial anisotropy is at an optimal angle (8°) to the SAW propagation. A high sensitivity of 630.4 kHz/Oe was measured, showing a capability of detecting vector magnetic fields [249].

SAW-based magnetic field sensors are a very active research area with many developments in the last 15 years. Currently, an impressive LOD of 10 pT/Hz^1/2^ has been demonstrated at 100 Hz, with a bandwidth of up to 1 MHz [250]. The aim is to further reduce LOD into the femto-tesla range, which is pivotal to bio-medical applications. However, to achieve this, there are still some challenges to specifically reduce the 1/*f* noise at low frequencies. Going forward, this noise can be reduced by achieving precise control of the magnetic layer (by using materials with a high piezomagnetic coefficient (CoFeC [142] or FeGaC [145] or materials with low losses [251,252,253]) and using piezoelectric materials with high electromechanical coupling and low dielectric and mechanical damping (LiNbO_3_ and LiTaO_3_, which have large piezoelectric coefficients compared to quartz, Table 2).

## 4. Magnetoelectric Antennas

### 4.1. BAW ME Antenna

Antennas are an integral part of electrical circuits that enable the transfer of information. Conventionally, the antennas are made from conductors, have sizes that are comparable to the electromagnetic wavelength λ_0_ of the frequency signal they are transmitting and can be scaled down at maximum to λ_0_/10 [254]. This size is too large for certain applications, where the space is limited, e.g., in mobile phones and medical devices. Moreover, conventional antennas, when mounted parallel to the ground surface, suffer from the ground plane effect, which results in a reduced antenna gain. In such applications, magnetoelectric (ME) antennas are very attractive alternatives. As such, antennas operate at their acoustic resonance, which is determined by their physical size and acoustic velocity, rather than their electromagnetic resonance. The acoustic wavelength is five orders of magnitude smaller than the electromagnetic wavelength at a given frequency. Additionally, the ME antennas do not suffer from the ground plane effect, and the in-phase magnetic image current further enhances the antenna gain by 3 dB [19].

The active component of a bulk acoustic wave resonator is a ME composite, which consists of a piezoelectric and magnetostrictive material. The piezoelectric layer of a ME antenna is sandwiched between the top and bottom electrode, while a magnetostrictive layer is deposited on top of the sandwiched stack. The resonator can function as an antenna in the following way. As a transmitter, the RF signal is fed to the piezoelectric layer via top and bottom electrodes. The signal appropriately deforms the materials via piezoelectric effect, and the strain is transmitted to the magnetostrictive material through the coupling between the two materials. The magnetostrictive material oscillates accordingly due to inverse magnetostriction (Villari effect) and radiates electromagnetic waves. Alternatively, the resonator functions as a receiver as a high-frequency magnetic field is radiated on top of the magnetostrictive layer, which deforms due to the magnetostriction effect. The strain of the magnetostrictive layer is transferred to the piezoelectric layer and generates the proportional voltage due to the inverse piezoelectric effect. The BAW ME antennas can primarily be classified into two different types based on their operation configuration, namely (1) thin-film bulk acoustic resonators (FBARs) and (2) solidly mounted resonators (SMRs).

#### 4.1.1. FBAR

**Figure 16:**
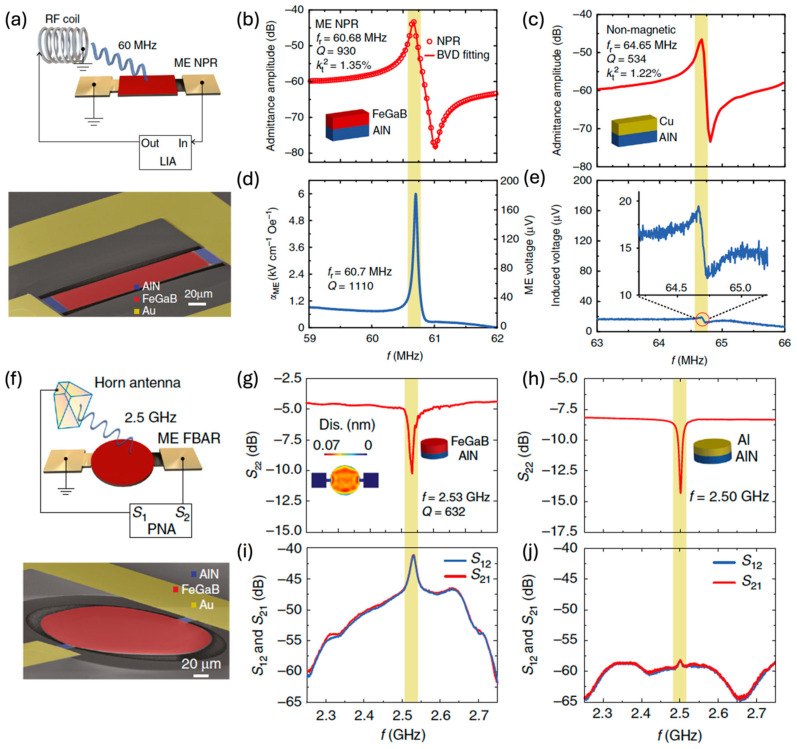
(**a**) The illustration of the NPR antenna and the measurement setup. The high-frequency magnetic field is generated by the RF coil. On the bottom, a SEM picture of the antenna is shown with different individual layers (AlN, FeGaB, and Au). (**b**) The admittance curve and the Butterworth–van Dyke mode, and the inset shows the various parameters. (**c**) Admittance curve of a control device, where the magnetic layer is replaced with Cu. (**d**) The ME coefficient (right) and ME-induced voltage in the piezoelectric layer as a function of frequency. (**e**) Analog-induced voltage in the piezoelectric layer of the control device; the inset shows a zoomed-in view of the red circle. (**f**) The set-up of a FBAR antenna that uses a horn antenna to excite the magnetostrictive layer. At the bottom, a SEM picture of the close-up of the FBAR antenna highlighting the individual layers (AlN, FeGaB, and Au). (**g**) Return loss curve (S_22_) of the ME FBAR antenna. The inset shows the simulated displacement of the ME FBAR device at resonance. (**h**) Return loss curve (S_22_) of the control FBAR device when the magnetic layer is replaced with a non-magnetic Al layer. (**i**) The transmission (S_12_) and receiving (S_21_) behavior of the FBAR antenna. (**j**) Transmission (S_12_) and receiving (S_21_) curves of the control FBAR device; note the sharp reduction of the peaks compared to the ME FBAR. Reprinted from Ref. [15].

Strain-mediated BAW-based ME antennas were first theoretically reported by Yao et al. [255] and Chen et al. [256]. The authors report a novel strain-mediated antenna that has a better radiation performance compared to conventional antennas. Experimentally, MEMS-based FBAR ME antennas were first reported by Nan et al. in 2017 [15]. Two different resonators were proposed, including a nanoplate resonator (NPR) which has a rectangular structure (oscillating along the width), and a FBAR (oscillating along the thickness). Both of the resonators consist of a piezoelectric AlN layer and a magnetostrictive FeGaB layer. Air cavities are formed below the resonators by etching substrates, in order to have excellent acoustic localization. Such a design, though challenging to fabricate, allows for better oscillation of the resonator. The resonance frequency of the NPR is 60.5 MHz, as determined by the width of the NPR. A RF coil was used to excite the antenna at its resonant frequency (Figure 16a). The NPR structure demonstrated α = 6 kV/cm Oe with an induced peak voltage of 180 μV. A control device was also demonstrated where the magnetostrictive FeGaB layer was replaced with Cu. This showed a peak-induced voltage of 18 μV, which is a factor of 10 below the ME NPR counterparts (Figure 16b–e).

The FBAR antenna demonstrated a clear resonance peak at 2.53 GHz (Figure 16f,g). The frequency is much higher compared to the NPR, as it is resonating along the thickness, which is one order of magnitude smaller compared to the width of the NPR. The ME FBAR antenna showed a gain of −18 dBi calculated by the gain transfer method. The authors compare the resonator performance with a control device, where the FeGaB layer is replaced with Al while keeping the rest the same. The control sample shows almost no transmission peaks, as is evident in the S_21_ and S_12_ peaks (Figure 16i,j). Thus, the transmission seen in the ME antenna directly results from the magnetic component (FeGaB).

**Figure 17:**
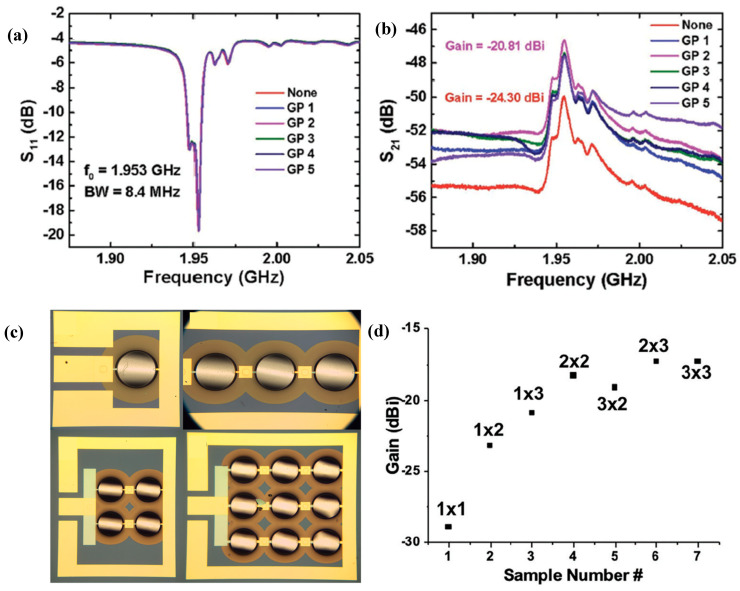
(**a**) Return loss curve (S_11_) of the ME FBAR antenna with a resonant frequency of 1.95 GHz and an 8.4 MHz bandwidth mounted on different sizes of ground planes (GP1–GP5). (**b**) Transmission (S_21_) curves of the ME FBAR antennas along with a control device (mounted on top of a 2 cm × 2 cm plastic substrate). Note the 3 dB gain enhancement of the ME FBAR mounted on GP vs. control device. (**c**) Optical images of four ME FBAR antenna array configurations. (**d**) Gain enhancement of different antenna configurations, showing a non-linear increase in gain as a function of antenna number. Reprinted from Ref. [19].

Recent efforts to increase the gain of the FBAR antennas were demonstrated by using an antenna array [19]. This work also highlights ground plane immunity and a 3 dB gain enhancement when the ME FBAR antennas were mounted on top of ground planes of various sizes (GP1-GP5, Figure 17a,b). Typically, when conventional antennas are placed close to or in-plane with large conductive substrates, they suffer from the ground plane effect, leading to suboptimal antenna performance. This is primarily due to the generation of image currents in the ground plane that are out of phase, which greatly degrades the antenna’s radiation efficiency. In contrast, ME antennas rely on magnetic radiation and generate in-phase image currents instead of out-of-phase image currents, which results in a 3 dB gain enhancement, regardless of the ground plane size (GP1–GP5).

The study investigated different ME antenna arrays that have different topologies including 1 × 1, 1 × 2, 1 × 3, 2 × 2, 3 × 2, 2 × 3, and 3 × 3 (Figure 17c). Increasing the number of antennas proportionally increases the volume of the magnetostrictive material and the total available magnetic moment for radiation. The authors expected that, as the number of antennas increased, the gain, radiation, and bandwidth would improve, since different structures have slightly varying thicknesses. The antennas consist of an AlN piezoelectric layer, with a multi-layer stack of FeGaB/SiO_2_ used as the magnetostrictive layer. The resonant frequency of the antennas in the array falls between 2.51 and 2.53 GHz, demonstrating excellent fabrication control.

The authors observed a non-linear increase in gain as a function of the antenna number (Figure 17d), mainly due to the mismatched input impedance as the resonator number increases. Nevertheless, the array with nine individual device elements showed the highest gain at −17.3 dBi, representing a 10 dBi enhancement compared to the single resonator. The radiation pattern of the ME antenna arrays follows that of a single ME antenna, with only the gain being enhanced while maintaining the same directionality. The increase in antenna size for the 3 × 3 array does not result in a proportional gain enhancement, indicating the need for further optimization. However, these efforts to realize such ME antenna arrays could enable multiband or wideband antennas for applications in wireless communication, power transfer, and implantable biomedical devices.

ME antenna bi-layer structures were also investigated for other applications. A NeuroRFID investigated by Zaeimbashi et al. consists of a compact ME antenna array with three NPRs that could simultaneously be used as energy harvesters and magnetic field sensors. The dual-band device has created a huge interest in the biomedical community [14]. The resonators have two resonating modes, one along the thickness and the other along the width of the oscillator. Like the previous FBAR antennas, an air gap between the substrate and the resonator is created to allow for strong acoustic wave excitation. As the thickness of the AlN is 500 nm, which is significantly lower than its width of 50 μm, it has a higher resonance frequency in the thickness mode at 2.5 GHz compared to the width mode at 63.6 MHz. The thickness mode was used for energy harvesting and showed a FOM of 3721, and the width mode was used for magnetic field sensing and allowed for a LOD of 470 pT/Hz^1/2^ (for details please check NPR array ME sensor section). Other devices such as a low-frequency MEMS-based ME magnetic sensor operating at 224 Hz were investigated. The resonating structure is a 70 μm diameter disk. The devices show a LOD of 800 pT at 1 μV at a resonance frequency of 224 kHz [257].

#### 4.1.2. SMR

The SMR antennas differ from FBAR antennas by having no air gap between the resonator and the substrate. In the FBAR operation, acoustic localization is achieved by the air gap. However, in the case of the SMR, a so-called Bragg reflector is used to achieve the localization. The Bragg reflector consists of alternating layers of materials with high and low acoustic impendence and acts as an acoustic mirror. The antennas are fabricated on top of the Bragg reflector to isolate the resonator during excitation. The SMR has multiple advantages compared to the FBAR, such as no need to release the resonator in fabrication and increased device robustness.

**Figure 18:**
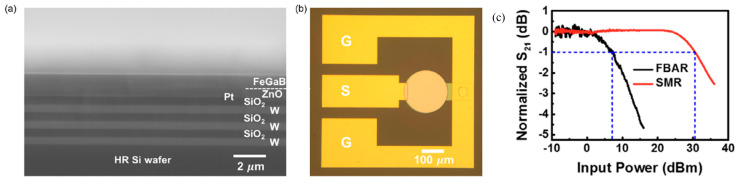
(**a**) Cross-sectional view of the SMR ME antenna showing the different layers. The Bragg reflector (3 × W/SiO_2_), Pt (bottom electrode), ZnO, and FeGaB layers are evident. (**b**) Optical image of the SMR ME antenna. The GSG pads are used to feed the signal during transmission mode or read out the signal in the receiving mode of the antenna. (**c**) The power-handling capability of the SMR ME antenna compared to the FBAR ME antenna. The SMR has a 1 dB compression point at 30.4 dBm while the FBAR has it at 7.1 dBm. Reprinted from Ref. [20].

The concept of SMR-based ME antennas was first proposed by Liang et al. in 2020 [16] and was more elaborately investigated in 2023 (Figure 18) [20]. The antennas were fabricated on top of a Bragg reflector (layered stack of W and SiO_2_). A SEM cross-section reveals the different layers of the antenna (Figure 18a,b). A highly textured ZnO with a Full Width Half Maximum (FWHM) of 2.29 was used as a piezoelectric material, while a 10-layer stack of (FeGaB (45 nm)/SiO_2_ (5 nm)) was used as a magnetostrictive layer. The antennas were excited with the help of a high-frequency magnetic field H_rf_ from a horn antenna along different directions. The maximum gain of −18.8 dBi is demonstrated when the H_rf_ is parallel to the easy axis direction, and it shows the lowest gain when the H_rf_ is parallel to the hard axis of the antenna. The authors also show an increased power-handling capability, with the 1 dB compression point at 30.4 dBm for the SMR, compared to 7.1 dBm for the FBAR counterpart (Figure 18c). Such high power-handling capabilities would allow SMR antennas to be supplied with higher input power, which could allow for the generation of stronger electromagnetic (EM) waves. A similar SMR-based antenna was reported by Ma et al., who demonstrated a SMR antenna working at 4.97 GHz with −25.1 dBi gain capable of 5G communication [258].

Over the last seven years, after the initial experimental evaluation of the BAW FBAR antennas, there have been significant breakthroughs in the field of ME antennas. The current research has been established for making antenna arrays with increased gain and SMR-based robust devices with high power-handling capability. However, for further enhancement of the antenna’s properties, two approaches can be considered. (1) A more detailed understanding of the micromagnetic dynamics driven by acoustic wave excitation must be realized, in particular, the correlation between the magnetic noise and the antenna’s properties during transmission and receiving modes. If the magnetic noise plays a major role, this can be reduced by incorporating exchange bias layers in the magnetic field layer. (2) Using materials with low losses can enhance the efficiency of the antennas. It is well demonstrated in the literature that AlN is a CMOS-compatible piezoelectric layer that has very low losses (tan δ: 0.025% ± 0.011, [75]). On the other hand, typical magnetostrictive materials have magnetic damping on the order of 10^−4^–10^−3^. Replacing them with yttrium iron garnets (which have a very low damping in the order of 10^−5^ [259]) would increase the radiation efficiency.

### 4.2. SAW ME Antenna

**Figure 19:**
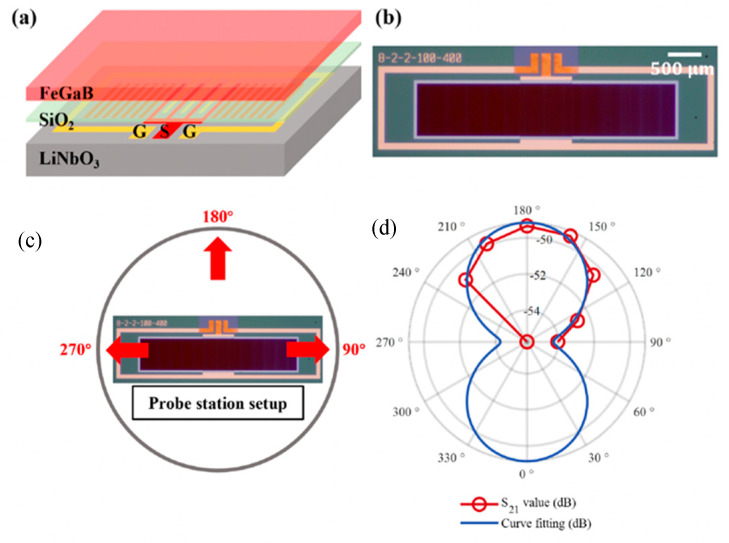
(**a**) The illustration of the SAW-based ME antenna showing different layer configurations. (**b**) The optical image of the SAW-based ME antenna. (**c**,**d**) Device testing schematic and the associated radiation pattern of the SAW ME antenna. Reprinted with permission from Ref. [261]. Copyright 2024 IEEE.

SAW-based ME antennas are a relatively new area of focus with only a couple of studies published to our knowledge [260,261]. A great advantage of using such SAW-based devices is that they can operate in the medical implant communication services (MICS) band (402–405 MHz), which is predominately used for implant communication. BAW-based NPR antennas that can operate in the MICS band were investigated previously, but the resulting structures are 8 μm wide, which are challenging to fabricate [262]. Realizing such 400 MHz FBAR antennas is challenging, as the thickness needed for a piezoelectric layer to excite is approximately 14 μm, which is challenging to achieve using physical vapor deposition techniques, such as sputtering or evaporation. Hence, SAW-based antennas provide an attractive alternative. In a recent 2024 study, Zhang et al. [261], showed a SAW-based antenna fabricated on top of a LiNbO_3_ substrate with FeGaB as the magnetostrictive component. As shown in Figure 19a–d, the antenna has (measured from the image 5 mm × 1.5 mm, [261]) increased in area by a factor of 16 compared to the bulk acoustic resonator (BAR) (SMR—0.67 mm × 0.67 mm, measured from [20]) counterparts. The proposed design showed an increased gain of −28 dBi at 430 MHz while using a FeGaB layer of 670 nm. Another new method was proposed to enhance the radiation of a SAW-based antenna operating at 1.87 GHz. The authors report an enhancement of efficiency by 70.95 ± 6.4% when a magnetic field was applied [260].

SAW-based ME antennas are relatively new, and there have been limited investigations. Their large size in comparison with BAW ME antennas gives them a relative disadvantage. More considerations into device miniaturization and increased gain must be taken.

### 4.3. Very-Low-Frequency Antennas

Very-low-frequency (VLF: 3–30 kHz) electromagnetic (EM) waves can penetrate dense conductive media such as earth and water, which makes them ideal for applications such as underwater communications and navigation, subterranean mapping, underground communication, and ionospheric remote sensing [19]. However, the long wavelength (10 km~100 km) of VLF signals requires antennas to be large and have high power consumption. The development of magnetoelectric (ME) antennas based on bulk and surface acoustic wave resonators has significantly reduced VLF antenna size, scaling down from several square kilometers to just a few square centimeters [263,264,265].

**Figure 20:**
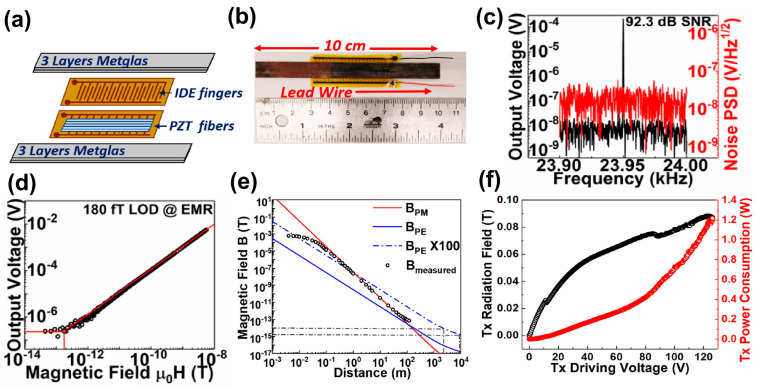
The VLF ME antenna: (**a**) A 3D model showing each layer. (**b**) An optical top view photograph with the antenna’s dimensions. (**c**) The measured received signal at the resonance frequency along with the noise floor. (**d**) The measured output voltage as the bias field decreases, showing the limit of detection. (**e**) Predicted and measured magnetic field distribution as a function of distance. (**f**) Radiation field and power consumption of the ME transmitter under varying driving voltages. Reprinted with permission from Ref. [263]. Copyright 2020 IEEE.

The VLF ME antenna concept was first put forward by Sun and Li [266] in 2016, and a practical device was reported by Dong et al. in 2020 [263]. A communication system operating at very low frequency (VLF) that employs a magnetoelectric (ME) transmitter alongside a ME receiver was demonstrated, utilizing a pair of ME heterostructures functioning at their electromechanical resonance (EMR) frequency. The structure and image of the device are depicted in Figure 20a,b, respectively. This VLF antenna features Metglas as its ferromagnetic component and lead zirconate titanate (PZT-5) as the piezoelectric element. By applying an electric field to the PZT fibers’ surface, a surface acoustic wave (SAW) is generated, with the resonance frequency being determined by the pitch of the interdigitated electrode (IDE) fingers. The magnetic domain’s rotation, controlled by ME coupling, acts as the radiation source for the antenna. A notable peak at 23.95 kHz was observed, with a signal-to-noise ratio (SNR) of 92.3 dB measured, as illustrated in Figure 20c. The system attained a low limit of detection (LOD) of 180 fT, indicating a high electric field sensitivity suitable for long-range communication (Figure 20d,e). The expected magnetic field produced by the ME transmitter is shown in Figure 20e and modeled through a near-field electric and magnetic dipole approach for the piezoelectric and magnetostrictive phases. With a LOD of 260 fT/Hz^1/2^, a communication range reaching up to 120 m was accomplished [263]. It is anticipated that a range between 2.5 and 10 km could be achievable with a compact array consisting of 100 elements. Lastly, Figure 20f reveals that, as the driving voltage increased from 0.5 to 120 V, there was a rise in both the radiation intensity and the power consumption. The power consumption reached approximately 300 mW when the voltage reached 60 V. Despite this advancement in VLF communication, the bandwidth and efficiency of VLF ME antennas are still constrained by relatively low resonance frequencies at VLF. The authors also introduce a direct antenna modulation (DAM) by using a non-linear response. With a carrier signal at EMR frequency and a magnetic modulation signal frequency of 100 Hz, the antenna achieved 29 dB SNR at a 16 m communication distance [263].

In 2022, Dong enhanced the magnetoelectric (ME) antenna by incorporating a higher Q-factor mechanical resonator using Metglas/PZT-8/Metglas, and by increasing the number of ME antennas in an array, he achieved a total radiation field of 200 nT at 1 m using 12 antenna array [264]. In the same year, Hu et al. developed a VLF antenna with a Metglas/PMN-PT structure, obtaining a giant converse magnetoelectric coefficient of 6 Oe·cm/V at 6.3 kHz [267]. In 2023, Du et al. demonstrated the feasibility of using ME antennas for portable underwater communication, reaching a maximum underwater propagation distance of 2.2 m [268]. Also in 2023, Fu et al. introduced a bias-free VLF ME antenna with the structure of annealed Metglas/Metglas/PZT/Metglas/annealed Metglas, which exhibited a high direct magnetoelectric coefficient (α_DME_ = 107 V/cm·Oe) and a converse magnetoelectric coefficient (α_CME_ = 9 G/V) without the need for biased magnetic field [269].

## 5. Magnetoelectric BAW/SAW Devices for Non-Reciprocity and NV^−^ Center Excitation

### 5.1. Magnetoacoustic SAW Non-Reciprocal Isolators

Recent advances in integrated nonreciprocal components, such as isolators and circulators, have enabled a gamut of wireless communication/sensing modalities that are otherwise not possible [270,271,272,273,274,275,276,277]. For instance, isolators can be used to protect on-chip high-power amplifiers from back reflection. Integrated circulators in conjunction with self-interference (SI) cancellation technology can enable the feasibility of in-band full-duplex operation for future wireless systems and networks. Conventional circulators are based on the constructive or destructive interferences of electromagnetic signals under magneto-optical Faraday rotation in ferrite materials at different signal ports [278]. The constructive interference at the first port in one circuitry direction and the destructive interference at the second port enable the functionality of signal transmission along one circuitry direction and forbid the other direction [278]. The isolators can be easily realized by adding a perfectly matching load at one of the circulator ports. These ferrite-based isolators/circulators exhibit low insertion loss (<1 dB) and a high power-handling capability of up to tens of watts [279]. However, the operation requirement of a kOe-level bias field necessitates the setup of strong permanent magnets or electromagnets, which make the commercial isolators/circulators bulky and expensive, with high power consumption, manufacturing complexity, and potential electromagnetic interference. The extremely high growth temperature of thick ferrite material makes the fabrication of these isolators/circulators incompatible with semiconductor manufacturing techniques.

Emerging efforts in non-magnetic integrated CMOS isolators/circulators have shown great potential for full-duplex transceivers based on circulators and SI cancelers for wireless applications [278,279]. However, the non-magnetic integrated CMOS isolators/circulators exhibit high DC power consumption, ranging from tens to hundreds of milliwatts. For instance, recently proposed non-magnetic CMOS circulators [270,271,275,276] based on transistor modulation suffer from high power consumption (40–200 mW), making them non-amenable for low-power applications. Additionally, current integrated CMOS nonreciprocal components exhibit low bandwidths due to their reliance on resonant ring structures [270,271,275,276]. However, when bandwidth is prioritized, their power handling is compromised [276,280]. Therefore, integrated nonreciprocal technology targeting low-power applications while exhibiting integrated high-efficiency, high-linearity, and high-isolation does not exist today and is of great importance for the future IoT-driven world.

In recent years, magnetoacoustic isolators have been widely investigated, which exhibit substantial nonreciprocity with remarkable power efficiency and CMOS compatibility, showing great potential for low-power and wideband full-duplex wireless radio systems. Magnetoacoustic non-reciprocal isolators comprise a magnetic stack between two interdigital transducers on a piezoelectric substrate. By applying a RF voltage on the transmitting IDT, a surface acoustic wave can be generated and propagate toward the other IDT. When the surface acoustic wave passes through the magnetic stack, it can interact with the non-reciprocal spin wave (SW) in the magnetic stack via various mechanisms, including magnetoelastic coupling [281,282,283], magnetorotation coupling [284,285,286], spin-rotation coupling [287,288,289], and gyromagnetic coupling [290]. Among them, magnetoelastic coupling and magnetorotation coupling are strong and widely used for magnetoacoustic isolators. Magnetoelastic coupling refers to the change of magnetization precession under a strain or vice versa, while magnetorotation coupling arises from reorientating magnetic anisotropy and dipolar shape anisotropy during SAW-driven lattice rotational motion. The interaction between SAW and non-reciprocal SW generates magnetoacoustic waves that exhibit a much higher backward loss rate than the forward one or vice versa. The dramatic difference between the damping rate of the forward and backward magnetoacoustic waves allows the signal transmission in one direction and dampens that in the other direction, contributing to a magnetoacoustic isolator.

Ongoing research strives to enhance non-reciprocity strength and bandwidth while maintaining high transmission between device ports. Even though the SAW-SW coupling is inherently non-reciprocal due to helicity mismatch between fixed counterclockwise magnetization precession and reversible lattice rotational motion chirality [284,291,292], this non-reciprocity is typically weak and less than 3 dB/mm [291,292,293] in a magnetic stack with reciprocal spin wave dispersive relation, such as a single magnetic film.

**Figure 21:**
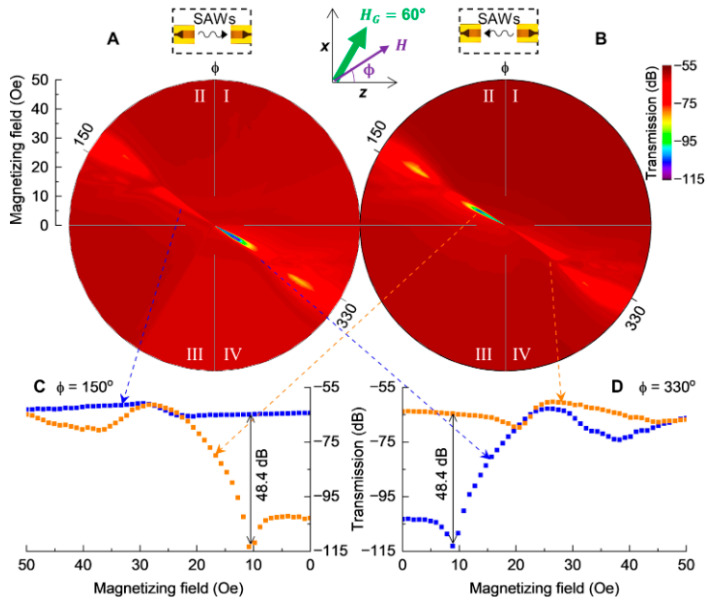
Experimental results on the giant nonreciprocity effect of hybridized SAW/SW in the FeGaB/Al_2_O_3_/FeGaB multilayer stack on the piezoelectric lithium niobate substrate at the frequency of 1435 MHz when a growth field (*H_G_*) was oriented at 60°. Acoustically Driven Ferromagnetic Resonance (ADFMR) plots were generated for hybridized SAW/SW traveling in the (**A**) +z direction and (**B**) −z direction. Resonance absorption, highlighted in blue on the color scale, was observed at the ADFMR frequency in directions orthogonal to the growth field, where strong magneto-acoustic interaction took place. (**C**) Field sweeps at *ϕ* = 150° for forward (blue) and reverse (orange) SAW propagation. (**D**) Field sweeps at *ϕ* = 330° for forward (blue) and reverse (orange) propagation. The isolation was determined by the difference between forward and reverse sweeps under identical static field conditions. The nonreciprocal power isolation of 48.4 dB was observed. Reprinted with permission from Ref. [294]. Copyright 2020 The American Association for the Advancement of Science.

Various magnetic stacks with non-reciprocal spin wave dispersion have been theoretically investigated and experimentally demonstrated, including interfacial Dzyaloshinskii–Moriya interaction (iDMI) stacks (e.g., CoFeB/Pt) [285], dipolar coupled stacks (e.g., FeGaB/SiO_2_/FeGaB) [294], and Ruderman–Kittel–Kasuya–Yosida (RKKY) synthetic antiferromagnets (e.g., CoFeB/Ru/CoFeB) [295], where the key parameters of the corresponding magnetoacoustic isolators are summarized in Table 4. The iDMI stack is typically composed of a magnetic layer and a heavy metal layer, such as a perpendicular magnetic anisotropy (PMA) Ta/CoFeB/MgO/Al_2_O_3_ stack [284]. The nonreciprocal behavior originates from iDMI and SAW-SW helicity mismatch (HM), the interaction between the chirality of rotational lattice distortions and the magnetization influenced by magnetic anisotropies. However, the bandwidth of such non-reciprocity is typically narrow (10~100 MHz) [296]. Non-reciprocity has also been demonstrated in interlayer dipolar coupling (IDC) stacks which are composed of two magnetic layers spaced by several nm thick insulating or conductive layers. As the spacer layer thickness decreases, the initially uncoupled reciprocal Damon–Eshbach spin wave (SW) modes in the two thin magnetic layers become coupled through the dipolar stray fields of the spin waves [297]. This coupling gives rise to symmetric (in-phase) modes and the antisymmetric (out-of-phase) modes [298,299]. The giant non-reciprocity of 48.4 dB/mm has been achieved at 1.435 GHz in the FeGaB (20 nm)/Al_2_O_3_ (5 nm)/FeGaB (20 nm) IDC stack with magnetization perpendicular to the non-collinear uniaxial anisotropy field directions (~60 degrees to the SAW propagation direction) [294], as shown in Figure 21. Phase non-reciprocity has also been realized at the same frequency and bias field condition [300].

**Figure 22:**
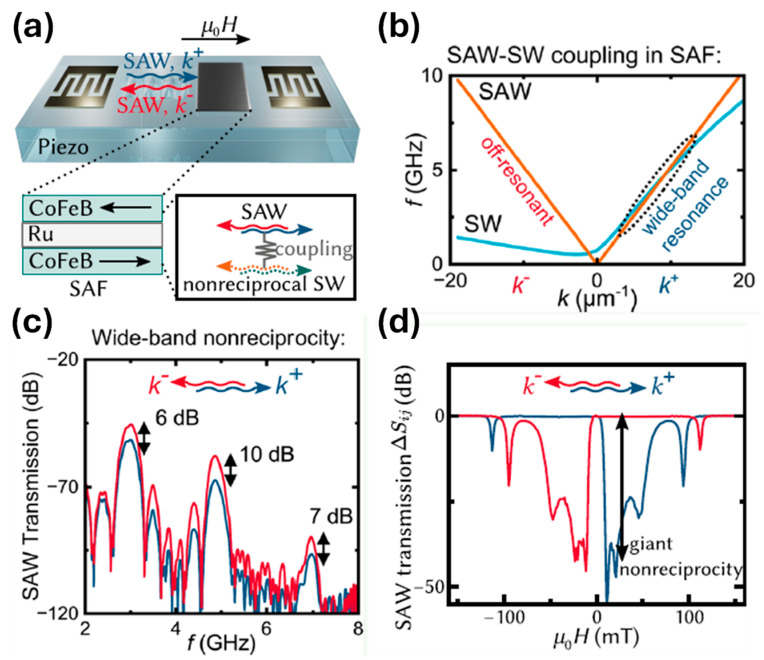
Wideband and giant nonreciprocity experimentally demonstrated in CoFeB/Ru/CoFeB RKKY stack. (**a**) Schematics of magnetoacoustic device. (**b**) SAW and SW dispersive relation near wide-band resonance region. (**c**) Wideband non-reciprocity from 2 to 7 GHz realized in CoFeB (20 nm)/Ru (0.46 nm)/CoFeB (20 nm) stack. (**d**) A 250 dB/mm giant non-reciprocity achieved in CoFeB (16 nm)/Ru (0.55 nm)/CoFeB (5 nm) stack. Image (**a**–**c**) is reprinted with permission from Ref. [304]. Copyright 2024 American Chemical Society; Image (**d**) is reprinted with permission from Ref. [295]. Copyright 2023 American Chemical Society.

For extremely thin (<1 nm) conductive spacer layers, it is necessary to account for interlayer RKKY exchange interactions [301,302]. RKKY antiferromagnetic exchange interaction has been theoretically proposed to have a wide non-reciprocal transmission band [296] and giant isolation strength suitable for wideband non-reciprocal RF isolators, circulators, and phase shifters [303]. Recently, this wide non-reciprocity has been experimentally demonstrated in a CoFeB/Ru/CoFeB RKKY stack [304], as shown in Figure 22a,b. A wideband non-reciprocity from 2 to 7 GHz was realized in the CoFeB (16 nm)/Ru (0.55 nm)/CoFeB (14 nm) stack, as shown in Figure 22c. The maximum non-reciprocity reached 10 dB for a 100 μm long magnetic stack at 4.86 GHz, corresponding to 100 dB/mm giant non-reciprocity. By optimizing the thickness of the CoFeB layer, 250 dB/mm giant non-reciprocity was achieved in the CoFeB (16 nm)/Ru (0.55 nm)/CoFeB (5 nm) stack [295], as shown in Figure 22d. Although synthetic antiferromagnetic (SAFM) structures have been quite successful, selecting a proper magnetic layer thickness is not trivial due to the trade-off between the IDC and RKKY requirements regarding the magnetic layer thickness. High IDC benefits from thicker magnetic layers or higher wavenumbers to enhance spin wave (SW) nonreciprocity. However, increasing the layer thickness weakens the RKKY antiferromagnetic coupling. Recently, the magnetoacoustic coupling between the shear horizontal waves in 36°-rotated or 42°-rotated Y-cut X-propagation LiTaO3 substrates and spin waves in ferromagnetic or anti-magnetostrictive bilayers has been investigated [305,306,307]. A giant non-reciprocity ranging from 60 dB/mm [305] to 82 dB/mm [307] has been realized when the bias field is applied perpendicular to the SAW propagation direction. The magnetoacoustic coupling in ferromagnetic bilayers [305] and anti-magnetostrictive bilayers [306,307] provides another practical and technologically simple system to realize transmission non-reciprocity for RF isolators/circulators.

As shown in Table 4, the demonstration of magnetoacoustic isolators typically yields a high insertion loss above 25 dB owing to high-order mode SAW harmonics, which is far from practical applications. Future work should be put into reducing insertion loss by utilizing fundamental mode IDT electrodes. In addition, a self-biased magnetic stack with a high magnetic anisotropy field should be developed to eliminate the need for a biased magnetic field, which necessitates the permanent magnet or electromagnet and makes the magnetoacoustic isolator bulky with high power consumption and electronic noise.

**Table 4:** Summary of the experimentally demonstrated non-reciprocal magnetoacoustic isolators.

Magnetic Stack	Piezoelectric Substrate	Mechanism	DC Bias Field $B_{0}$ (mT)	Operation Frequency f (GHz)	Magnetic Stack Length l (um)	Off Magnetoacoustic Resonance Insertion Loss ${IL}_{0}$ (dB)	On Magnetoacoustic Resonance Insertion Loss ${IL}_{m}$ (dB)	Non-Reciprocity (dB/mm)	Ref.
Ta/CoFeB(1.6 nm)	Y-cut Z-propagation LiNbO_3_	SAW-SW HM, iDMI	110	6.1	-	-	-	-	[284]
CoFeB(5 nm)/Pt	Y-cut Z-propagation LiNbO_3_	SAW-SW HM, iDMI	21	6.77	750	71	87.5	28	[285]
FeGaB(20 nm)/Al_2_O_3_(5 nm)/ FeGaB(20 nm)	Y-cut Z-propagation LiNbO_3_	IDC	1	1.435	2200	60	65	22	[294]
NiFe(20 nm)/Au(5 nm)/CoFeB(5 nm)	Y-cut Z-propagation LiNbO_3_	IDC	21	6.87	500	89	89.8	74	[297]
CoFeB(20 nm)/Ru(0.46 nm)/ CoFeB(20 nm)	128°-rotated Y-cut X-propagation LiNbO_3_	IDC, RKKY	5	1.4	1000	29	33	37	[308]
Pt/Co(2 nm)/Ru(0.85 nm)/Co(4 nm)/ Pt	Y-cut Z-propagation LiNbO_3_	IDC, iDMI, RKKY	60	6.77	750	81	81.75	3	[309]
CoFeB(16 nm)/Ru(0.55 nm)/ CoFeB(5 nm)	128°-rotated Y-cut X-propagation LiNbO_3_	IDC, RKKY	20	5.08	150	81	81.135	250	[295]
Ta(2 nm)/Ru(2 nm)/CoFeB(16 nm)/Ru(0.55 nm)/CoFeB(14 nm)/Si3N4(3 nm)	Y-cut Z-propagation LiNbO_3_	IDC, RKKY	13.8	3 4.86 6.96	100	45.6 57.8 90.3	45.6 57.8 90.3	60 100 70	[304]
FeCoSi-B(10 nm)/NiFeCu(10 nm)	36°-rotated Y-cut X-propagation LiTaO_3_ substrate	IDC	9	2.33	500	54	69	60	[305]
Ni (16 nm)/Ti (8 nm)/FeCoSiB(16 nm)/Ti (10 nm)	42°-rotated Y-cut X-propagation LiTaO_3_	IDC	5.7	2.333	500	53	55	82	[307]

### 5.2. Magnetoacoustic BAW/SAW Devices for NV^−^ Center Excitation

Negatively charged nitrogen-vacancy (NV^−^) centers in diamonds, comprising a nitrogen atom and a nearby lattice vacancy, are quantum spin defects with unique properties [310]. These centers exhibit extended coherence times exceeding 100 µs at room temperature [311], exceptional fidelity [312], and facile optical initialization and readout of quantum states [313]. Combined with their responsiveness to magnetic excitations over a wide temperature range, these features make NV^−^ centers highly promising for quantum computing, communication, and information-processing applications [314,315,316], as well as for use as non-invasive nanoscale magnetic sensors [317,318,319].

The coherent manipulation of NV^−^ centers can be achieved via alternating current (AC) magnetic fields, electric fields [320], and strain waves [321], enabling their function as hybrid quantum transducers and computational units. Unlike direct AC magnetic field excitation via microwave antennas, spin wave (magnon) excitations offer long-distance, coherent control of NV^−^ centers over scales ranging from hundreds of microns to millimeters. Moreover, magnons can deliver 100 times stronger local driving fields with identical power inputs [322,323,324]. This power-efficient approach minimizes off-resonant spin wave noise at high power [325] while preserving the long coherence times required for magnon-mediated entanglement and communication between NV^−^ center qubits. These properties facilitate the development of on-chip quantum processors and computational units. Furthermore, NV^−^ centers can couple to terahertz optical photons, supporting their potential as microwave-to-optical quantum transducers that convert GHz qubit excitations into terahertz photons [326] for long-distance quantum information transfer. Together, the magnon–NV^−^ and NV^−^–optic–photon couplings serve as foundational elements for hybrid quantum networks [327,328,329,330,331].

Phonons, with their extended lifetimes in solid-state systems, also represent excellent information carriers for quantum and coherent information processing. The small wavelengths of acoustic waves render phonons particularly suitable for on-chip quantum systems [332]. Strain wave-driven magnon excitations, which are more power-efficient than microwave-driven methods, can significantly reduce the size of NV^−^-center-based quantum transducers. In strong coupling regimes, magnon–phonon interactions lead to magnon–polaron hybrid states, enabling efficient information transduction between magnon and phonon systems. This process paves the way for phonon–magnon–NV–photon hybrid quantum transduction platforms.

**Figure 23:**
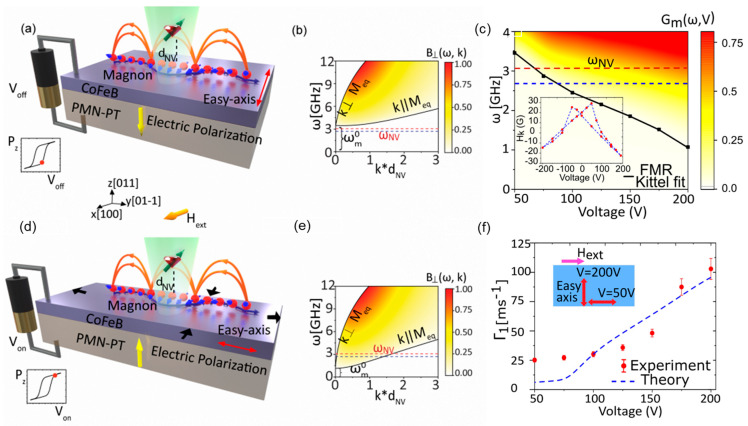
Magnetoelectric control of NV^−^ center by CoFeB/PMN-PT BAW resonator. (**a**,**d**) Schematic illustration of the quantum spin defects (QSD)-magnon hybrid sample fabricated by dispersing nanodiamonds containing ensembles of NV^−^ centers on a thin ferromagnetic film of CoFeB on a 300 μm thick PMN-PT ferroelectric substrate. (**b**,**e**) Maps of normalized *B* as a function of *ω*−*k* for both *V*_on_ and *V*_off_. The black lines enveloping the colormap are the calculated magnon dispersion lines for bulk modes (*k*∥M) and surface modes (*k*⊥M). The dashed colored lines represent the NV^−^ ESR lines *ω*_NV_. (**c**) FMR frequency ($\omega_{m}^{0}$) (data in black lines) as a function of applied voltage extracted from the experimental results for a fixed external magnetic field *H*_ext_ = 57 G along *x*-axis. The color map represents the calculated values of the magnetic noise spectral density *G*_m_(*ω*, *V*) for an effective NV^−^ height *d*_NV_ = 77 nm. The dashed colored lines represent the maximum spread of the NV^−^ ESR lines *ω*_NV_. The inset shows the detailed measurements of the magnetic anisotropy field as a function of applied voltage. (**f**) Measured relaxation rates Γ_1_ as a function of applied voltage for a fixed *H*_ext_ = 57 G along the *x*-axis. The inset shows a schematic illustration of the magnetic anisotropy field for the two different voltages for a fixed *H*_ext_. The dashed line represents the theoretical fit of relaxation rates Γ_1._ These figures are reproduced from Ref. [333].

Recent advancements have demonstrated the magnetoelectric control of NV^−^ centers through magnetoelectric coupling in CoFeB/300 µm PMN-PT heterostructures [333], as depicted in Figure 23. The application of an external voltage (V) to the PMN-PT substrate induces changes in its electrical polarization, which generates lattice strain. This strain is transferred to the CoFeB film, altering its magnetic anisotropy, as illustrated in Figure 23a,d. When a fixed external magnetic field (*H*_ext_) is applied along the [100] *x*-axis, increasing the voltage from *V* = *V_off_* to *V* = *V_on_* reverses the polarization (*P_z_*), tuning the magnon band into resonance with the NV^−^ electron spin resonance (ESR) transitions, as shown in Figure 23b,e. This magnon band shift arises from the coupling between electric, elastic, and magnetic orders within the multiferroic heterostructure. Specifically, as the voltage increases from 50 V to 200 V, the anisotropy field (*H*_k_) decreases consistently from *H*_k_ = 30 Gauss to *H*_k_ = −22 Gauss, signifying a flipping of the magnetic easy axis from the *x* to the *y* direction, accompanied by the reversal of *P_z_* (Figure 23c). Concurrently, the lowest spin relaxation rate (Γ_1_) increases significantly from 25.1 ± 2 [ms]^−1^ at *V* = *V*_off_ to 102.8 ± 9 [ms]^−1^ at *V* = *V*_on_, corresponding to a remarkable 400% tuning of Γ_1_. This pronounced tunability of Γ_1_ underscores the potential of magnetoelectric control to facilitate robust interactions between magnons and NV^−^ centers in a highly energy-efficient manner.

**Figure 24:**
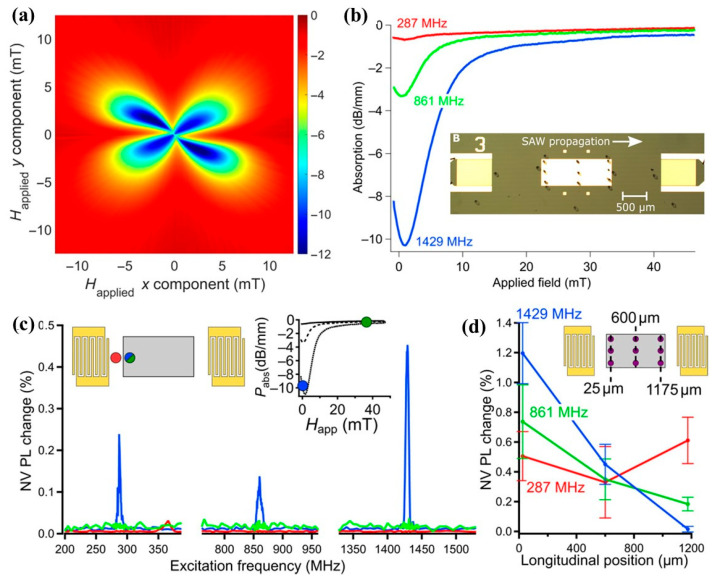
Energy-efficient and local control of NV^−^ centers by SAW-driven magnon resonance. (**a**) Plot of power absorption as a function of applied magnetic field for a 20 nm nickel ADFMR device at 1429 MHz. The x component of the field is taken to be parallel to the direction of SAW propagation, and the y component is in-plane and perpendicular to the direction of SAW propagation. The color bar indicates absorption in decibels per millimeter. (**b**) Line-cut along the angle of highest absorption (45°) showing a large field-dependent attenuation at 287, 861, and 229 MHz. The insets show the photograph of IDTs, magnetoelastic film, and clusters of nanodiamonds on the measured device. (**c**) Change in PL normalized to the DC level for NV^−^ centers located off the ferromagnetic pad (red), and NV^−^ centers on the pad with zero field (blue) and a high (35.8 mT) applied bias field (green) (**d**) NV^−^ PL change in a 20 nm nickel sample as a function of longitudinal position from the edge of the ferromagnet closest to the excitation IDT at zero applied magnetic field. These figures are reproduced with permission from [334]. Copyright 2018 The American Association for the Advancement of Science.

Surface acoustic wave (SAW) resonators are particularly advantageous for fostering strong magnon–phonon interactions, as they efficiently concentrate acoustic energy on the surface, facilitating robust coupling with spin waves. Furthermore, their planar structures simplify the fabrication process compared to bulk acoustic wave resonators. Recent advancements in SAW-driven magnon resonance have demonstrated highly localized and efficient control of NV^−^ centers, achieving Rabi frequencies comparable to those induced by microwave excitation but with over 1000 times greater power efficiency [322,323]. In a key experiment, a 20 nm Ni or Co film was positioned between interdigital transducers (IDTs) operating at a fundamental frequency of 287 MHz [334]. The resulting power absorption exhibited a fourfold symmetry, peaking at *ϕ* = 45 degrees, as illustrated in Figure 24a. This absorption could be tuned using a biased magnetic field applied at 45 degrees, as shown in Figure 24b. At the fifth harmonic frequency of 1429 MHz, the power absorption exceeded that observed at both the fundamental and third harmonic frequencies. At this 1429 MHz frequency, a direct photoluminescence (PL) change in NV^−^ centers was detected, aligning closely with their excited state resonance frequency (Figure 24c). The observed PL change is attributed to ferromagnetic resonance (FMR), as no PL signal was recorded at high fields near the IDT. The intensity of the NV^−^ PL change diminished with the increasing distance from the leading edge of the magnetic film, which is consistent with signal absorption as the excitation propagates through the magnetic layer, as depicted in Figure 24d. To enhance the performance of magnetoelectric SAW (ME SAW) devices, it is imperative to utilize magnetic materials with low damping and strong magnetoelastic coupling. Additionally, the confinement and focus of surface acoustic wave energy are crucial for achieving efficient and localized control of NV^−^ centers via SAW-driven magnetic resonance. Recent innovations include the application of Gaussian focusing techniques in SAW devices, which suppress acoustic diffraction loss and significantly enhance the quality factor (Q) to 22,400 at cryogenic temperatures (~30 K) [335]. Moreover, Bragg reflectors have been employed to enable strong magnon–phonon coupling, achieving a cooperativity of 1.2 [336]. Advancing the acoustic wave confinement and focusing strategies represents a promising research direction. Such efforts will support the development of strong magnon–phonon interactions and enable the coherent control of NV^−^ centers, paving the way for hybrid quantum transducers that integrate magnons, phonons, NV^−^ centers, and photons.

## 6. Conclusions and Outlook

Based on a magnetostrictive/piezoelectric composite, BAW and SAW ME devices have revolutionized sensing, RF, and quantum technologies. BAW and SAW ME devices enable multiple functionalities, including ultra-sensitive magnetic sensing, high-gain wireless communications, extremely efficient wireless power transfer, non-reciprocal RF isolation and circulation, and energy-efficient excitation of quantum defects.

Over the past decade, ME sensors have garnered significant attention as promising tools for magnetic sensing applications. Utilizing magnetic/electrical frequency conversion and the delta-E effect, intensive efforts have been put into applying novel piezoelectric or magnetostrictive materials or stacks, improving piezoelectric or magnetostrictive material properties, electrode or sensor structure designs, and identifying and reducing thermal, magnetic [337], and acoustic noise [338]. Tens of pT magnetic noise performances have been realized in the low-frequency band for detecting bio-magnetic signals. Thin-film ME sensors offer low-profile, high spatial resolution, and contactless measurement options compared to electrode-based systems. Demos in magnetocardiography (MCG) and magnetic particle mapping (MPM), magnetoneurography (MNG), and magnetomyography (MMG) have been developing [7,8,14,15,339,340,341,342]. However, a sensitivity gap still exists between thin-film ME sensors and leading magnetometers, like SQUIDs and optically pumped magnetometers (OPMs). To narrow this gap, it is crucial to develop low-noise, soft magnetostrictive thin films with giant piezomagnetic coefficients and piezoelectric materials with a high mechanical quality factor [343], since the SNR of ME sensors in resonance mode increases by Q_m_^1/2^ and the SNR increases by Q_m_^3/2^ for the delta-E operation scheme [233,242,343]. In addition, acoustic mode optimization is also important. It has been demonstrated that second-order bending modes increase magnetic sensitivity compared to first-order modes due to the lesser impact of inhomogeneous internal stray fields and the weighting of local properties [344]. ME resonators with innovative structures and vibration modes at higher frequencies and high-quality factors can significantly boost signal-to-noise ratios (SNR). Controlling intrinsic noise in ME sensors also requires carefully managing magnetic domain activity, which can be achieved through engineering the magnetic layers and optimizing the excitation fields. Novel acoustic noise rejection and signal-processing techniques should be developed to reduce the need for bulky acoustic shielding chambers and reduce electronic noise, which is now limiting the performance of ME sensors. ME sensor array technology should also be investigated with effective crosstalk and interference rejection to increase screen time and ensure data accuracy and diagnosis reliability. Next-generation ME sensors should aim to reach detection limits in the femto-Tesla range across a wider frequency range (from 0.01 Hz to 1 kHz), which is achievable with innovative ME sensor designs and advanced noise-cancellation techniques.

Compared to conventional electrical antennas, whose sizes are limited by electromagnetic wavelength in meter-level at GHz frequencies, ME antennas exhibit an ultra-compact ,and ground plane immunity and 3 dB gain enhancement owing to magnetic dipole radiation. FBAR, SMR, and SAW ME antennas with different resonator structures and BAW/SAW acoustic modes have been demonstrated, with advancement in antenna gain, power-handling capability, radiation efficiency, and ground plane immunity. ME antennas with multiple functional bands for wireless communication, energy harvesting, and magnetic field sensing have been developed. Future work should be put into ME antenna array technologies to further increase the antenna gain and widen the bandwidth with ME resonator disks with different resonance frequencies. In addition, magnetic stacks with high self-biased resonance frequencies and piezoelectric materials with high electromechanical coupling should be integrated to realize strong magnon–phonon coupling at a zero-bias field, which has been theoretically demonstrated to boost antenna gain, radiation efficiency, and bandwidth [345]. To realize this, low-damping magnetic materials should be used in ME antennas with a CMOS-compatible integration method. For instance, integrating YIG films via ion implantation [346,347,348,349] onto piezoelectric layers should be explored for developing energy-efficient ME antennas.

Wideband and giant non-reciprocity have been demonstrated in magnetoacoustic devices where the Rayleigh surface acoustic wave interacts with non-reciprocal spin waves in magnetic stacks, including interfacial DMI stacks (e.g., CoFeB/Pt) [285], dipolar-coupled stacks (e.g., FeGaB/SiO_2_/FeGaB) [294], and RKKY synthetic antiferromagnets (e.g., CoFeB/Ru/CoFeB) [295]. Future efforts need to be put into the investigation of non-reciprocal spin waves with other SAW modes, like Sezawa mode [350], reducing the insertion loss by fundamental mode SAW, self-biased non-reciprocity without the need for external magnets, and system design of multiple magnetoacoustic devices, to realize wideband non-reciprocity from 2 to 16 GHz.

Efficient control of quantum defects such as NV^−^ centers has been realized in BAW and SAW devices with 1000 times less power than microwave excitation. Future research should aim to achieve a heterogeneous integration of low-loss magnetic materials in ADFMR devices to facilitate NV^−^ center manipulation within diamond crystals. This approach seeks to preserve the spin wave amplitude and propagation distance while enhancing the relaxation time (T_1_) and coherence time (T_2_) of the NV^−^ centers and ensure that quantum information, initially transferred from acoustic phonons and stored in magnons, maintains sufficient longevity without significant decay or loss due to relaxation or decoherence. Extended T_1_ and T_2_ ensure the robust amplitude and extended propagation distance of spin waves and enable the long-distance transmission of quantum information across diverse storage and processing platforms through magnons. Fundamental mode SAW near 2.87 GHz should be utilized to excite spin waves in a magnetic stack for the excitation of NV^−^ center ground states, which have a much longer lifetime than the excited states. A careful design should be conducted to ensure that magnetoacoustic devices have strong absorption at the frequency and magnetic field that align with the spin resonance of NV^−^ centers.

## Figures and Tables

**Table 2:** (**a**) Summarizing the important piezoelectric materials and their material properties used in SAW and BAW devices. (**b**) summarizing low-loss piezoelectric substrates for SAW and BAW devices.

(a)									
Material	Piezoelectric Coeff. (pC/N)		Relative Permittivity		Density (Kg/mm^3^)	Elastic Modulus (GPa)	tan⁡δ (%)	Electromechanical Coupling Efficiency $k_{t}^{2}$)	Comments and Main Applications
			$\varepsilon_{11}$	$\varepsilon_{33}$					
PZT	$d_{31}=$−243 [74]	$d_{33}$ = 574 [74]	2440 [74]	2870 [74]	7500 [3]	$C_{11}$$=C_{22}$$=127.2,\;C_{33}$$=117.4,\;C_{44}$$=C_{55}$$=23,\;C_{66}$ = 23.5 [3]	1.6 [74]	20–35% [74]	Highly used bulk and thin films (nano-rods) as sensors, actuators, energy harvesters, and antennas.
AlN	$d_{31}=$−2 [3]	$d_{33}$=3.5–4.96 [3]	9.2 [3]	10.1 [3]	3300 [3]	$C_{11}$$=C_{22}$$=410,\;C_{33}$$=389,\;C_{44}$$=C_{55}$$=125,\;C_{66}$ = 130, $C_{12}$$=C_{21}$$=149,\;C_{13}$$=C_{23}$$=C_{31}$$=C_{32}$ = 99 [3]	0.025 ± 0.011 [75]	Thin-film bulk acoustic resonator (FBAR): 7% [76]; contour mode resonator (CMR): 2% [77]; contour mode Lamb wave resonator (CMLR) resonator: 5.33%~7.1% [78,79,80]	CMOS compatible, lead-free with very low loss.
Al_1-x_Sc_x_N	$d_{31}=-2.32-15.90x+82.0x^{2}-243x^{3}\;$ [81]	$d_{33}=5.12+44.4x-253x^{2}+745x^{3}$ [81]	-	89.93x+10.31(1−x)−62.48x(1−x) [82]	3530 (x = 0.14), 3560 (x = 0.26) [81,83], $3.806x+3.255(1-x)-0.298x\left(1-x\right)$ [82]	$C_{11}(x)=410.2(1-x)+295.3x-210.3x(1-x)$$,\;C_{12}(x)=142.4(1-x)+198.6x-61.9x(1-x)$$,\;C_{13}(x)=110.1(1-x)+135.5x+78.9x(1-x)$$,\;C_{33}(x)=385.0(1-x)-23.8x-101.4x(1-x)$$,\;C_{44}\left(x\right)=122.9\left(1-x\right)+169.5x-137.3x\left(1-x\right)$ [84]	0.025~0.1 [85]	Lamb wave resonator: 7.83~10.28% [86,87]; contour mode resonator (CMR): 3.2%~5% [88]; Sezawa SAW mode: 3.8%∼ 4.5% [89]; Rayleigh SAW: 2%~2.2% [90]; Thin-film bulk acoustic resonator (FBAR): 5% ~14% [91]	CMOS-compatible, lead-free, promising ferroelectric material [92,93,94,95,96].
(PMN)_0.7_-(PT)_0.3_	$d_{31}$ = −1395	$d_{33}=2000$	4963	1386	7800~7820 [97].	$C_{11}$$=160.4,\;C_{12}=149.6,\;C_{13}$$=124,\;C_{33}$$=120,\;C_{44}$$=53,\;C_{66}$ = 28	<1	62%	Typically used in piezoelectric transducers, sonar systems, and energy harvesters. Material properties from [98].
(PZN)_0.92_–(PT)_0.08_	$d_{31}$ = −1250	$d_{33}$= 2500	2900	7700	8315	$C_{11}$$=115,\;C_{12}=105,\;C_{13}$$=109,\;C_{33}$$=115,\;C_{44}$$=63.4,\;C_{66}$ = 65	1–1.2	50%	Highest $d_{33}$ allows their use in ultrasonic devices in medical industry, actuators, and energy harvesters, among others. Material properties from [98]
ZnO	$d_{31}$ = −5 [99]	$d_{33}$ = 12.4 [99]	9.2 [3]	12.6 [3]	5665~5680 [100,101]	$C_{11}$$=206,\;C_{12}=118,\;C_{13}$$=118,\;C_{33}$$=211,\;C_{44}$ = 44 [102]	0.025~0.05 for RF sputtered thin films [103,104,105]. ALD film: 0.0001~0.002 [106]	ZnO/SiC SAW resonator: 1.5%~3% [107]; FBAR ZnO resonator: 0.5%~3.4% [101]; Lamb wave resonator: 12.4%~14% [108,109]	Lead free, bio-compatible, and non-toxic [110]
(**b**)									
**Material**	**Piezoelectric Coeff. (pC/N)**	**Relative Permittivity**		**Density** **(Kg/m^3^)**	**Elasticity** **(GPa)**	tan⁡δ (%)	**Orientation**	**Electromechanical Coupling Efficiency (** $k_{t}^{2}$ **)**	**Comments**
		$\varepsilon_{11}$ **=** $\varepsilon_{22}$	$\varepsilon_{33}$						
LiNbO_3_	$\begin{array}{c} d_{21}=-d_{22}=-21,\\ d_{31}=d_{32}=-1,\\ d_{33}=6,\\ d_{24}=d_{15}=68,\\ d_{16}=-42 \end{array}$ [3]	84 [3]	30 [3]	4650 [26]	$C_{11}$$=C_{22}$$=203,\;C_{33}$$=243,\;C_{44}$$=C_{55}$$=59.9,\;C_{66}$ = 74.8, $C_{12}$$=C_{21}$$=52.9,\;C_{13}$$=C_{23}$$=C_{31}$$=C_{32}$$=74.9,C_{14}=-C_{24}=C_{41}=-C_{42}=C_{56}=C_{65}=9$ [3]	0.002–0.004 [111]	41° Y cut-X propagating	Shear-horizontal SAW: 33.54% [112]	High electromechanical coupling, low dielectric and acoustic loss, used for SAW sensors, resonators, and filters [113]
							X-cut Y propagating	coupled shear mode surface acoustic wave (CS-SAW) on LiNbO_3_/SiC: 34% [114]	
							128° Y cut-X propagating	Rayleigh SAW: 5.5% [113]; first-order antisymmetric (A1) Lamb mode: 46.4% [115]	
LiTaO_3_	$\begin{array}{c} d_{21}=-d_{22}=-7,\\ d_{31}=d_{32}=-2\\ d_{33}=8,\\ d_{24}=d_{15}=26,\\ d_{16}=-14 \end{array}$ [3]	51 [3]	45 [3]	7465 [26]	$C_{11}$$=C_{22}$$=233,\;C_{33}$$=275,\;C_{44}$$=C_{55}$$=94,\;C_{66}$ = 93, $C_{12}$$=C_{21}$$=46.9,\;C_{13}$$=C_{23}$$=C_{31}$$=C_{32}$$=80.2,\;C_{14}=-C_{24}=C_{41}=-C_{42}=C_{56}=C_{65}=-11$ [3]	0.06 [116]	Z cut	Longitudinal BAW: 2.7% [117]	Used as a substrate material for BAW and SAW devices [118]; Used for shear horizontal wave based magnetoacoustic non-reciprocal RF devices
							X cut, 112.2°–Y propagating	FBAR: 17.4% [117]; Fast shear BAW: 21.6% [117]	
							36 ° Y Cut–X propagating	Longitudinal BAW: 9.9% [117]; Leaky-SAW (L-SAW): 5.7% [119]	
							42° Y Cut X–propagating	Longitudinal BAW:9.0% [117]; shear-horizontal SAW (SH-SAW) on LiTaO_3_/SiC: 5.58% [120]	
Quartz	$d_{11}$= 2.31, $d_{14}$= 0.727 [118]	4.52 [118]	4.68 [118]	2560 [26]	$C_{11}$$=87.26,\;C_{33}$$=105.8,\;C_{44}$$=57.15,\;C_{66}$ = 40.35, $C_{12}$$=6.57,\;C_{13}$$=11.95,\;C_{14}=-17.18$ [121]	0.01 [122,123]	ST-cut (42°75′ Y-cut, X-propagating)	Calculated for temperature sensor 0.14% [124]	Low acoustic loss, and high resonant frequency stability over a broad range of temperatures and pressures, used for BAW oscillators (typically AT-cut quartz with thickness shear mode) for frequency control in communication systems and clocks and SAW filters (ST-cut) [118]
							AT-quartz (35°15′ Z-cut, X-propagating)	Bulk acoustic wave devices 8.8% [125]	

**Table 3:** List of magnetostrictive materials in the literature. Also given are the saturation magnetostriction and the piezomagnetic coefficient.

	$\lambda_{s}$ (ppm)	$\left(\frac{\partial \lambda }{\partial H}\right)$/d_33, m_ (ppm/Oe)	Crystallography	Fabrication Method	Thermal Stability (°C) *	Comments/Ref.
Terfenol-D Tb_0.27_Dy_0.73_Fe_2_	1840	2.4	Single crystal, bulk	Casting	650	highest $\lambda_{s}$ [133,138,139]
Galfenol (Fe_81_Ga_19_)	395	3	Single crystal, bulk	Casting	700	[134,140]
FeSiBMn (2605SA1 Metglas, Inc)	27	-	Amorphous, ribbons	Melt spinning	395	Low loss, high permeability [135,141]
(Fe_90_Co_10_)_78_Si_12_B_10_	30	6.3	Amorphous, thin film	Magnetron sputtering	-	Amorphous thin film [10]
(Co_50_Fe_50_)_95.2_C_4.8_	60	10.3	Amorphous, thin film	Magnetron sputtering	500	[142]
(Fe_81_Ga_19_)_88_B_12_	75	7	Amorphous, thin film, as deposited	Magnetron sputtering	-	[143,144]
(Fe_81_Ga_19_)_88_B_12_	75	12	Amorphous, thin film, annealed @280°C for 120 min	Magnetron sputtering	-	Highest piezomagnetic coefficient [140]
(Fe_80_Ga_20_)_89_C_11_	81.2	9.71	Amorphous, thin film	Magnetron sputtering	-	[145]

* Thermal stability can either be Curie temperature or in the case of amorphous materials, the temperature the material forms nano-crystalline order, thus reducing its magnetostriction.
